# Diet-Induced Nutritional Stress and Pathogen Interference in *Wolbachia*-Infected *Aedes aegypti*

**DOI:** 10.1371/journal.pntd.0005158

**Published:** 2016-11-28

**Authors:** Eric Pearce Caragata, Fernanda Oliveira Rezende, Taynãna César Simões, Luciano Andrade Moreira

**Affiliations:** 1 Grupo Mosquitos Vetores: Endossimbiontes e Interação Patógeno Vetor, Centro de Pesquisas René Rachou—Fiocruz, Belo Horizonte, Minas Gerais, Brazil; 2 Serviço de Apoio a Métodos Quantitativos, Centro de Pesquisas René Rachou—Fiocruz, Belo Horizonte, Minas Gerais, Brazil; Instituto Oswaldo Cruz, BRAZIL

## Abstract

The pathogen interference phenotype greatly restricts infection with dengue virus (DENV) and other pathogens in *Wolbachia*-infected *Aedes aegypti*, and is a vital component of *Wolbachia*-based mosquito control. Critically, the phenotype’s causal mechanism is complex and poorly understood, with recent evidence suggesting that the cause may be species specific. To better understand this important phenotype, we investigated the role of diet-induced nutritional stress on interference against DENV and the avian malarial parasite *Plasmodium gallinaceum* in *Wolbachia*-infected *Ae*. *aegypti*, and on physiological processes linked to the phenotype. *Wolbachia*-infected mosquitoes were fed one of four different concentrations of sucrose, and then challenged with either *P*. *gallinaceum* or DENV. Interference against *P*. *gallinaceum* was significantly weakened by the change in diet however there was no effect on DENV interference. Immune gene expression and H_2_O_2_ levels have previously been linked to pathogen interference. These traits were assayed for mosquitoes on each diet using RT-qPCR and the Amplex Red Hydrogen Peroxide/Peroxidase Assay Kit, and it was observed that the change in diet did not significantly affect immune expression, but low carbohydrate levels led to a loss of ROS induction in *Wolbachia*-infected mosquitoes. Our data suggest that host nutrition may not influence DENV interference for *Wolbachia*-infected mosquitoes, but *Plasmodium* interference may be linked to both nutrition and oxidative stress. This pathogen-specific response to nutritional change highlights the complex nature of interactions between *Wolbachia* and pathogens in mosquitoes.

## Author Summary

Mosquito-transmitted disease severely impacts human health around the world. One novel form of control involves infecting medically important mosquito species with the naturally occurring bacterium *Wolbachia*, which restricts dengue and malaria transmission through a process called pathogen interference. The interference phenotype is still poorly understood, and potentially involves multiple physiological changes to the mosquito. We examined the role of nutritional stress on pathogen interference in the dengue vector *Aedes aegypti*, in order to better understand factors that might lead to variable interference. We demonstrated that interference against malaria-causing *Plasmodium gallinaceum* was dependent on mosquito nutritional status, however interference against dengue was not, implying that pathogen interference operates differently for different pathogens. We then examined mosquito immune processes that had been previously correlated with pathogen interference, and demonstrated that mosquito nutrition did not affect the expression of key mosquito immune genes, but did affect levels of reactive oxygen species. Our results highlight the complexity of the phenotype, and importantly suggest that adult nutrition may not be a key determinant of interference against DENV.

## Introduction

*Wolbachia pipientis* (Rickettsiaceae) is an obligate bacterial endosymbiont that shows great potential as a natural control agent for a range of clinically important mosquito-transmitted pathogens, including those responsible for diseases such as malaria and dengue in humans [1, 2]. *Wolbachia* naturally infect an estimated 40% of terrestrial insect species [3]. Infection often results in manipulation of host biology, with the nature and extent of these manipulations varying depending on the host and infecting *Wolbachia* strain [4, 5].

*Wolbachia* are maternally transmitted, and heavily infect host ovaries. The bacterium is often associated with extreme manipulation of the host reproductive process, furthering bacterial propagation at the expense of host fitness [6]. These manipulations allow the bacterium to naturally spread into uninfected insect populations, and to move across large distances [7]. The most common reproductive manipulation is cytoplasmic incompatibility (CI). CI-causing *Wolbachia* strains prevent or restrict viable egg production when uninfected females mate with *Wolbachia*-infected male insects, while *Wolbachia*-infected females can successfully breed with either infected or uninfected males. Infection can affect other host physiological processes including oogenesis [8], chemosensory perception [9] and parasitism [10]. Some strains form mutualistic relationships with their hosts, contributing resources [11], or enhancing fitness [12], while others are metabolically dependent on their hosts, and the resources they consume [13, 14].

*Wolbachia* naturally infect many mosquito species including *Aedes albopictus* and *Culex pipiens*, but not the primary dengue vector *Aedes aegypti* or most anopheline vectors of human malaria. Infections in *Ae*. *aegypti* have been generated in the laboratory via transinfection [15], through the injection of cytoplasm from the eggs of a *Wolbachia*-infected donor species into *Ae*. *aegypti* embryos [16–18]. These infections cause pathogen interference [19, 20], a *Wolbachia*-induced decrease in susceptibility to infection with pathogens including the dengue (DENV), chikungunya, yellow fever and West Nile viruses, filarial nematodes and some bacteria [17, 21–24]. Pathogen interference can result in decreased pathogen load, and largely prevent disseminated viral infection and salivary transmission [17, 25, 26]. Interference against DENV has been thoroughly studied in *Ae*. *aegypti* infected with the *w*Mel and *w*MelPop *Wolbachia* strains, with the strength and prevalence of the interference phenotype dependent on the viral isolate and serotype [25].

*Wolbachia* can also interfere with *Plasmodium* infection in mosquitoes, however interaction between the bacterium and these parasites appears to be more variable. The only stable *Wolbachia* transinfection in an anopheline mosquito, *w*AlbB in *Anopheles stephensi*, reduced *Plasmodium falciparum* oocyst and sporozoite numbers [27]. Infection with *w*MelPop in *Ae*. *aegypti* produced stronger interference against *P*. *gallinaceum* [23]. However, this effect may not be representative of how *Wolbachia* interacts with *Plasmodium* species that infect humans, given that the *Plasmodium* species that infect different animals are phylogenetically distinct [28], and that there are genetic, metabolic and immunological differences between anopheline and culicine mosquitoes [29, 30]. Prior to transinfection, *Wolbachia* infection in anophelines was studied using transient infection via somatic injection of *Wolbachia*. Some of these associations produced pathogen interference, however for *w*AlbB infections of *Anopheles gambiae*, *Plasmodium berghei* infection was enhanced [31]. This enhancement may be temperature dependent [32], and has also been observed for some native *Wolbachia* infections, including in *Culex pipiens* where *Wolbachia* protects the host against *Plasmodium*-induced mortality, but also increases susceptibility to infection [33–35]. Interestingly, such enhancement has never been observed for *Plasmodium* species that infect humans, or in a mosquito with a stable *Wolbachia* transinfection.

The process underlying pathogen interference remains poorly understood, while potential causes of enhancement are only hypothetical [31, 36]. Strong pathogen interference is typically associated with high *Wolbachia* density [17, 37]. The effect has been linked to activation of immune effector genes [22, 23, 38, 39], increased induction of reactive oxygen species (ROS) and related genes, which serve as part of the host defence against pathogens [38, 40], and competition for host cholesterol in *Drosophila melanogaster* [41]. Critically, none of these effects occur universally amongst the species and *Wolbachia* strains where pathogen interference has been observed, which suggests that the underlying mechanism may be complex, and that it could potentially be dependent on the length of the host-symbiont relationship [39, 42, 43].

Pathogen interference and CI serve as the basis for a form of *Wolbachia*-dependent mosquito control through mosquito population replacement [2, 44], which is currently being utilised for *Ae*. *aegypti* and dengue (www.eliminatedengue.com). This involves the release of *Wolbachia*-infected mosquitoes that mate with the wild population, where CI increases the *Wolbachia* infection frequency over successive generations [45]. High prevalence of pathogen interference in these mosquito populations would then potentially reduce disease transmission amongst humans [25]. Successful *Wolbachia* invasion is dependent on local environmental conditions, and a high proportion of infected individuals [44, 46]. Another critical factor is the competitiveness of released mosquitoes [47], with high fitness costs, as seen with the *w*MelPop strain [16, 48, 49], leading to rapid loss of infection in field populations [50]. In contrast, the *w*Mel strain has minimal fitness costs [17], and a stably infected population has persisted for several years in the field [51], with no loss of pathogen interference observed since the initial release [52].

Nutritional status and diet are key factors in an insect’s ability to resist infection with a pathogen [53–55]. Likewise, many pathogens are dependent on host nutritional resources, and can manipulate host metabolic process in order to facilitate infection [56–59]. Recent evidence has demonstrated that *Wolbachia* has a similar metabolic dependency [41, 60, 61], and this suggests that there is great potential for tripartite interactions between *Wolbachia*, pathogens, and host metabolism and nutrition to play a role in pathogen interference.

To that end, we have used dietary carbohydrate concentration as a platform to study the influence of host nutrition on the complexity and plasticity of pathogen interference and associated processes in female *w*Mel-infected *Ae*. *aegypti*. We investigated the role of diet-induced nutritional stress on interference against DENV and *P*. *gallinaceum*, and levels of immune gene expression and H_2_O_2_, which have previously been linked to the phenotype. Through these experiments we sought to further understanding of how *Wolbachia* can influence pathogen infection.

## Results

### DENV infection

**Fig 1:**
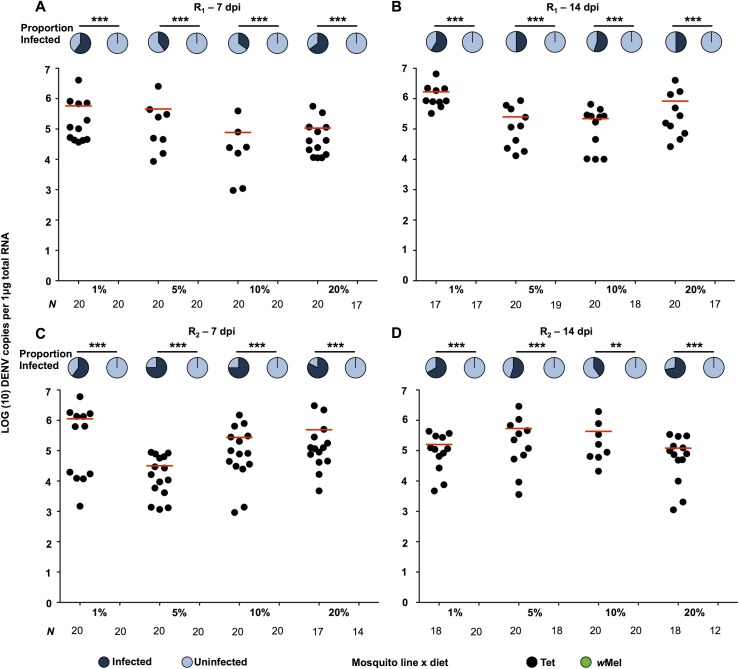
Interference against DENV-3 in *w*Mel-infected *Ae*. *aegypti* fed on different carbohydrate regimes. DENV-3 prevalence and intensity data for *w*Mel (+*Wolb*) and Tet (-*Wolb*) mosquitoes fed on one of four carbohydrate diets after experimental oral infection (R_1_—**A** - 7dpi, **B** - 14dpi; R_2_—**C** - 7dpi, **D** - 14dpi), as determined by RT-qPCR quantification using a DENV-specific TaqMan probe. Pie charts represent prevalence of infection (dark blue—proportion infected, light blue—proportion uninfected), and dot plots represent viral load in infected mosquitoes. Horizontal lines in each treatment represent mean viral load. *P* values: ** < 0.01, *** < 0.001, Prevalence—Fisher’s exact test.

In all experiments described below, adult mosquitoes were fed one of four carbohydrate regimes (1%, 5%, 10% or 20% raw sugar solution). Two experimental infections with a recently circulating Brazilian DENV-3 isolate were performed to determine if altered carbohydrate diets affected pathogen interference against DENV. Mosquitoes were fed on the carbohydrate regimes for 7 days post-eclosion, and then orally challenged with DENV. In both replicates, no DENV RNA was amplified from any *w*Mel sample at either 7 (Fig 1A & 1C) or 14 days post-infection (Fig 1B & 1D), for any diet. In contrast, the Tet infection rate varied between 35% and 82%, depending on diet, and the duration of infection. Prevalence (proportion infected with DENV-3) was consequently significantly higher for Tet mosquitoes than for *w*Mel, for each diet (Fisher’s exact test; *P* = 0.0033 - <0.0001). As no *w*Mel mosquitoes became infected, only the viral load data for Tet mosquitoes were compared statistically. There was a significant difference in viral load due to host nutrition at 7 dpi for both replicates, characterized by higher DENV levels on the 1% diet (Kruskal Wallis; R_1_—*P* = 0.0489; R_2_—*P* = 0.0084). At 14 dpi there were higher DENV levels on the 1% diet in the first replicate (Kruskal Wallis; *P* = 0.0015), but no effect in the second replicate.

### *Plasmodium gallinaceum* infection

**Fig 2:**
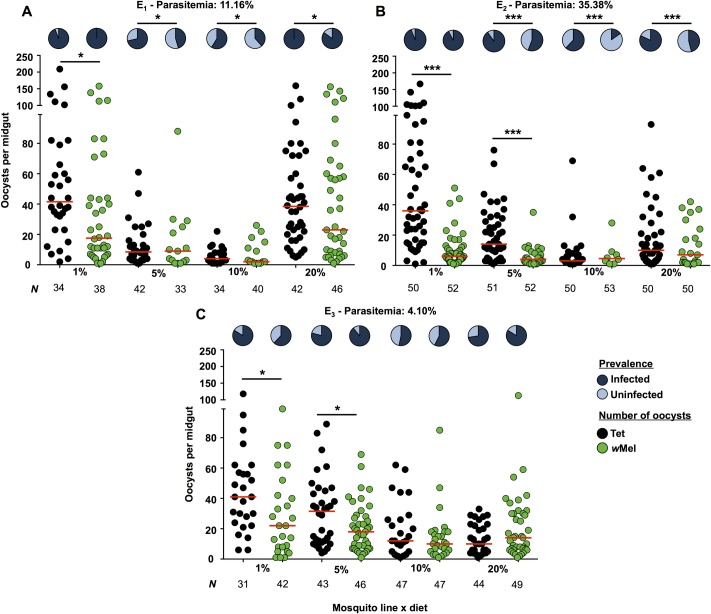
Interference against *P*. *gallinaceum* in *w*Mel-infected *Ae*. *aegypti* fed on different carbohydrate regimes. In three experimental replicates (E_1_—**A**, E_2_—**B**, E_3_—**C**), *w*Mel (+*Wolb*) and Tet (-*Wolb*) mosquitoes were fed one of four different carbohydrate regimes and then fed on a single *Plasmodium gallinaceum*-infected chicken. Prevalence of infection was determined by counting the proportion of mosquito’s that had oocysts in their midguts at 7–8 days post-infection (pie charts: proportion infected—dark blue, proportion uninfected—light blue). Intensity of infection was calculated as the number of oocysts per midgut for Tet (black circles), and *w*Mel mosquitoes (green circles). Red bars represent median oocysts for *P*. *gallinaceum*-infected mosquitoes. *P* values: * < 0.05, *** < 0.001, Prevalence—Fisher’s exact test, Intensity—Mann Whitney *U* test.

Three replicate *P*. *gallinaceum* infection experiments were performed to assess the impact of dietary carbohydrate levels on the ability of the *w*Mel *Wolbachia* strain to interfere with *Plasmodium* infection (Fig 2). Prevalence (proportion of mosquitoes infected) and intensity (number of oocysts in infected mosquito midguts) of *Plasmodium* infection were measured at 7–8 days post-infection, and data were compared independently for each experiment using binomial regression to determine the effects of *Wolbachia* infection and diet (S1 Table). In both experiment 1 (Fig 2A) and 2 (Fig 2B), *Wolbachia* was a significant factor affecting prevalence (Binomial models; E_1_ -, *P* = 0.0014; E_2_—*P* < 0.0001). There was a strong inhibitory effect of *w*Mel on the 10% diet in both experiments, however this attenuated as dietary carbohydrate levels changed, as evidenced by a significant effect of diet (Binomial models comparing each diet against the 10% diet; E_1_−1% diet and 20% diet—*P* < 0.0001; E_2_−1%, 5% and 20% diets—*P* < 0.0001). Interestingly, pairwise comparisons of prevalence for *w*Mel and Tet mosquitoes on each diet revealed that some level of interference was maintained for all diets except the 1% (Fisher’s exact test; E_1_—*P* < 0.05, E_2_—*P* < 0.001).

In experiment 3 (Fig 2C), the prevalence of *Plasmodium* infection observed for the 10% diet was greater than in the other two experiments, although the parasitemia level was lower. In this experiment there was no overall effect of *Wolbachia* on prevalence (Binomial regression; *P* = 0.646), however there was still a significant effect of diet (Binomial models; 1% diet—*P* < 0.05: 5% and 20% diets—*P* < 0.001).

*Plasmodium* intensity data were compared independently for each experiment using binomial negative regression, and we observed a significant effect of *Wolbachia* infection only in experiment 2 (Binomial negative regression against 10% diet; *P* < 0.0001). As for prevalence, a change in diet led to increased intensity of infection for all three experiments when compared to the 10% diet (Binomial negative regression: E_1_, E_2_—All diets: *P* < 0.0001, E_3_−1% and 5%: *P* < 0.0001, 20%: *P* > 0.05, all comparisons in reference to the 10% diet). However, pairwise comparisons for each diet indicated that a significant interference effect due to *Wolbachia* was induced only for low carbohydrate diets, as *w*Mel infection reduced the intensity of infection on the 1% diet in all 3 experiments (Mann Whitney U test; E_1_—*P* = 0.0151; E_2_—*P* < 0.0001; E_3_—*P* = 0.0231), and for the 5% diet in experiments 2 and 3 (Mann Whitney U test; E_2_—*P* < 0.0001; E_3_—*P* = 0.0376).

### Longevity assay

To obtain a broad indicator of fitness changes due to host nutrition, we compared the effects of altered dietary carbohydrates on the longevity of *Wolbachia*-infected *w*Mel mosquitoes using Cox Regression. Average mosquito survival was greater with higher dietary carbohydrate levels (S1 Fig). Average (± s.e.m.) survival time on the control diet was 29.48 ± 1.15 days, which was 12.28 days and 4.26 days longer than the average survival times for mosquitoes reared on the 1% and 5% diets, but 5.57 days shorter than the average for the 20% diet. Diet was a significant factor affecting mosquito longevity (Cox Regression; *P* < 0.0001). The 1% (*B* = 4.41, 95% CI = 3.32–5.88, *P* < 0.0001) and 5% (*B* = 1.38, 95% CI = 1.06–1.79, *P* = 0.016) regimes were associated with significantly higher hazard ratios than the control diet (10%), however the 20% diet led to a lower hazard ratio than the control (*B* = 0.60, 95% CI = 0.46–0.78, *P* < 0.0001).

### *Wolbachia* levels

**Fig 3:**
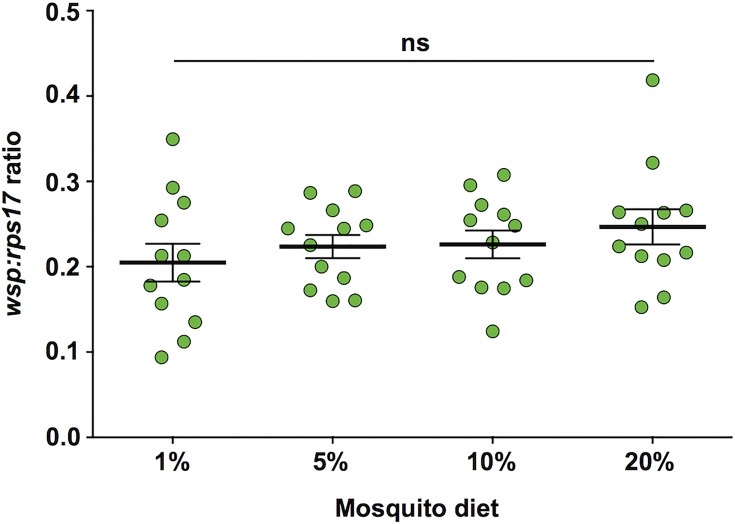
*Wolbachia* levels in *w*Mel-infected *Ae*. *aegypti* fed on different carbohydrate regimes. Expression levels of *Wolbachia surface protein* (*wsp*) were quantified relative to the mosquito *rps17* gene for 12 pairs of female mosquitoes from each diet using RT-qPCR. Each circle represents one pair of mosquitoes. Solid black lines represent mean expression (± s.e.m.). *P* values: ns > 0.05

Expression levels of the *Wolbachia* gene *wsp* were quantified relative to the host *rps17* in mosquitoes after 7 days of feeding on the different dietary regimes, in order to determine if the different regimes altered *Wolbachia* levels in *w*Mel mosquitoes (Fig 3). These data were compared statistically using univariate general linear models, which indicated that there was no statistically significant effect of diet (GLM; *P* = 0.468).

### Immune gene activation

We looked at whether changing dietary carbohydrate levels affected the expression of four genes associated with immune activation by *Wolbachia*.

**Fig 4:**
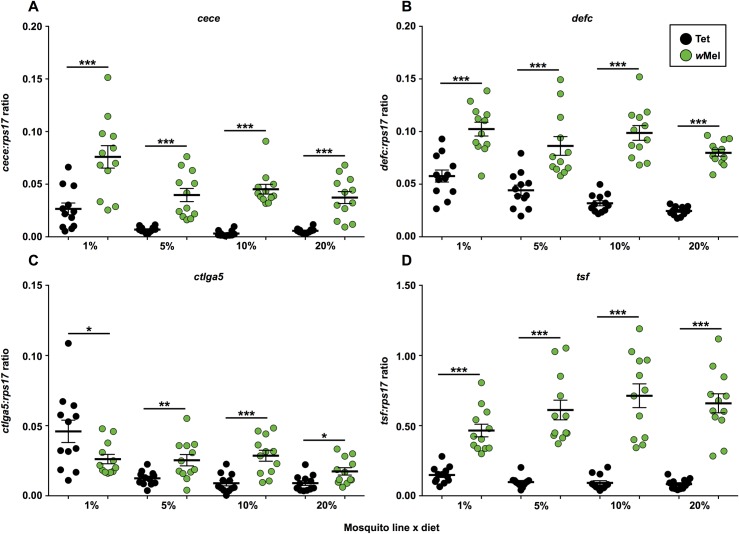
Immune activation in *w*Mel-infected *Ae*. *aegypti* fed on different carbohydrate regimes. Levels of 4 key immune genes, Cecropin E (*cece*) **(A)**, Defensin C (*defc*) **(B)**, C-type lectin, galactose binding 5 (*ctlga5*) **(C)**, and Transferrin (*tsf*) **(D)**, were quantified through RT-qPCR for *w*Mel (+*Wolb*) and Tet (-*Wolb*) mosquitoes, after 7 days feeding on their respective carbohydrate diets. Expression levels were normalized against host *rps17* expression levels. Each circle represents one pair of either Tet (black circles) or *w*Mel (green circles) mosquitoes, with 12 samples examined for each treatment. Solid black lines represent mean expression (± s.e.m.). *P* values: Student’s *t* tests, * < 0.05, ** < 0.01 *** < 0.001.

These genes, Cecropin E (*cece*) and Defensin C (*defc*), both antimicrobial peptides stimulated by the Toll and IMD immune pathways, C-type lectin galactose binding 5 (*ctlga5*), a carbohydrate-binding protein involved in bacterial recognition, and Transferrin (*tsf*), an iron transport protein, were all strongly upregulated by *w*Mel infection in Australian mosquitoes [39]. Expression data for each gene were analysed independently using general linear models to determine if *Wolbachia* infection status or nutrition had a major effect (Fig 4). Data for all immune assays were obtained from mosquitoes fed on carbohydrates for 7 days. These mosquitoes were not blood fed or infected with a pathogen.

Levels of *cece* (Fig 4A) and *defc* (Fig 4B) were significantly affected by both *Wolbachia* infection and diet (GLM; *P* < 0.0001), however there was no effect of interaction between diet and *Wolbachia* infection. Analysis of individual treatments revealed that *cece* and *defc* levels were higher in *w*Mel mosquitoes than in Tet for all diets (Student’s *t* tests; *P* < 0.001). Levels of *cece* were increased on the 1% diet for both *w*Mel and Tet mosquitoes, while *defc* expression in Tet mosquitoes was increased on the 1% and 5% diets, although average expression levels were still lower than for *w*Mel.

*Wolbachia* did not have a significant effect on *ctlga5* expression in the overall GLM model, however both diet and the *Wolbachia* x diet interaction (GLM; *P* < 0.0001) were significant factors. Expression levels of *ctlga5* were higher in *w*Mel mosquitoes than in Tet for all diets except the 1% (Fig 4C; student’s *t* tests—5% & 10%; *P <* 0.01, 20%; *P* < 0.05), where levels in *w*Mel mosquitoes remained high, but Tet levels were slightly higher (Student’s *t* test; *P* = 0.0324). There was a decrease in expression for *w*Mel mosquitoes on the 20% diet, where levels were on average 39.06% lower than for the 10% diet.

Expression levels of *tsf* (Fig 4D) were not significantly affected by diet in the overall model, but were affected by both *Wolbachia* (GLM; *P* < 0.0001) and *Wolbachia* x diet (GLM; *P* = 0.015). *tsf* levels were significantly higher for *w*Mel than Tet for all 4 diets (Student’s *t* tests; *P* < 0.0001). However, *tsf* expression in *w*Mel mosquitoes on the 1% diet was significantly lower than for the 10% and 20% diets (Student’s *t* test; *P* < 0.05).

### Immune pathway regulation

The expression of 8 genes with putative regulatory roles in the mosquito IMD (*caspar* and *rel2*), JAK-STAT (*domeless* and *pias*), JNK (*ap-1* and *jnk*) and Toll (*cactus* and *rel 1A*) immune pathways was examined in order to determine whether diet x *Wolbachia* interactions had a broader effect on host immunity (S2 Fig). We observed no effect of *Wolbachia*, diet, or *Wolbachia* x diet interaction in the expression of these genes. The one exception to this was for *pias*, a putative negative regulator of the JAK-STAT immune pathway, where *Wolbachia* but not diet or the *Wolbachia* x diet interaction was a significant predictor in the overall model (GLM; *P* = 0.044). In biological terms, this translated to higher *pias* expression in *w*Mel mosquitoes compared to Tet, but only for the 10% diet.

### Stress response & ROS induction

**Fig 5:**
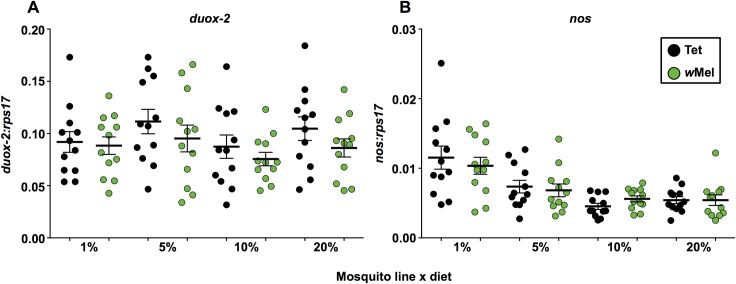
Levels of key oxidative stress response genes in *w*Mel-infected *Ae*. *aegypti* fed on different carbohydrate regimes. Levels of Dual Oxidase 2 (*duox-2*) **(A)** and Nitric Oxide Synthase (*nos*) **(B)** were quantified for Tet (-*Wolb*) and *w*Mel (+*Wolb*) mosquitoes using RT-qPCR. Gene expression values were normalized against host *rps17* expression. Each circle represents one pair of either Tet (black circles) or *w*Mel (green circles) mosquitoes, with 12 samples examined per treatment. Solid black lines represent mean expression (± s.e.m.). *P* values: General linear models, ** < 0.01, *** < 0.001.

Expression levels of *duox-2* (Fig 5A), an important gene in mosquito reactive oxygen species production, were unaffected by *w*Mel infection, diet, or diet x *Wolbachia* interaction (GLM; *P* < 0.05). Likewise levels of *nos* (Fig 5B), which is involved in nitric oxide production, were unaffected by the presence of *Wolbachia* (GLM; *P* < 0.05). However, we observed a significant increase in *nos* expression associated with lower carbohydrate diets in the overall model (GLM; *P* < 0.0001), and independently for both Tet (GLM; *P* < 0.0001) and *w*Mel mosquitoes (GLM; *P* = 0.001). Average *nos* levels were 45.92% higher for *w*Mel mosquitoes on the 1% diet than those on the 10% diet (student’s *t* test; *P* = 0.0013). For Tet mosquitoes, the 1% diet had on average 60.74% higher *nos* levels than the 10% diet (student’s *t* test; *P* = 0.0005), and those from the 5% diet had on average 38.56% higher *nos* levels (student’s *t* test; *P* = 0.0099).

**Fig 6:**
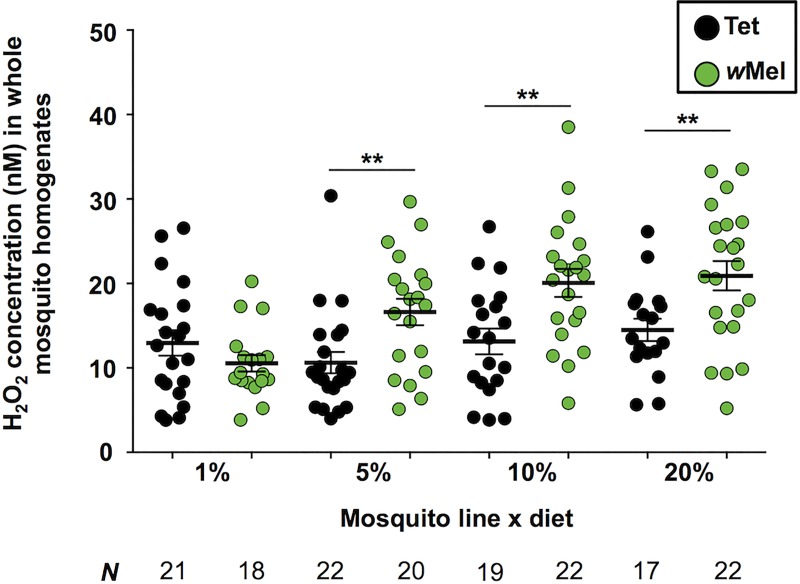
ROS induction in *w*Mel-infected *Ae*. *aegypti* fed on different carbohydrate regimes. Levels of reactive oxygen species (H_2_O_2_) were quantified for whole *w*Mel (+*Wolb*) and Tet (-*Wolb*) mosquitoes from all 4 diets using the Amplex Red Hydrogen Peroxide/Peroxidase Assay Kit. Each circle represents one pair of either Tet (circles) or *w*Mel (green circles) mosquitoes, with 17–22 samples examined for each treatment. Solid black lines represent mean expression (± s.e.m.). *P* values: Student’s *t* tests, ** < 0.01.

H_2_O_2_ levels were quantified in pairs of female mosquito after spending 7 days feeding on the different carbohydrate diets (Fig 6). *Wolbachia* infection (GLM; *P* < 0.0001), diet (GLM; *P* < 0.0001) and *Wolbachia* x diet (GLM; *P* = 0.0006) were all significant factors affecting H_2_O_2_ levels in mosquitoes. H_2_O_2_ levels in Tet mosquitoes did not change due to diet however mean H_2_O_2_ levels in *w*Mel mosquitoes were positively correlated with dietary carbohydrate concentration. ROS induction due to *Wolbachia* infection was observed for each of the three highest concentration diets, where significantly higher levels were observed in *w*Mel mosquitoes (Student’s *t* tests; 5% diet—*P* = 0.0046; 10% diet—*P* = 0.0040; 20% diet—*P* = 0.0082), however on the 1% diet there was no effect of *Wolbachia* (Student’s *t* test; *P* = 0.2034).

## Discussion

### A contrasting effect of host nutrition on DENV & *Plasmodium* interference

Pathogen interference in *Wolbachia*-infected *Ae*. *aegypti* restricts or prevents infection and transmission of DENV and other pathogens [22, 23, 25]. Interference is fundamental to transmission-blocking strategies that use *Wolbachia* to combat mosquito-transmitted disease [44], yet the underlying biological processes remain poorly understood. Competition for nutrients is important to interference in *Drosophila* [41], but no link with host nutrition had previously been made in *Ae*. *aegypti*. To that end, we fed *w*Mel (+*Wolb*) and Tet (-*Wolb*) *Ae*. *aegypti* mosquitoes with 1 of 4 carbohydrate diets (1%, 5%, 10% or 20% sucrose solution), and challenged them with either DENV-3 or *P*. *gallinaceum*.

We observed strong interference to both pathogens on the 10% (control) diet. For *P*. *gallinaceum*, *w*Mel infection reduced the prevalence of infection but did not affect intensity, while no *w*Mel mosquitoes became infected with DENV. Pathogen interference against *P*. *gallinaceum* had not previously been described for *w*Mel-infected *Ae*. *aegypti*. This effect was not as strong as for *w*MelPop-infected *Ae*. *aegypti* where there was greatly reduced prevalence and intensity of infection [23], although that strain has a higher bacterial density, which likely promotes stronger pathogen interference [17, 25]. Our DENV interference results were similar to results from other DENV isolates, where mosquitoes were reared on 10% sucrose [17, 25].

Altering host nutritional status by feeding 1%, 5% or 20% sucrose led to increased prevalence of *P*. *gallinaceum* infection in *w*Mel mosquitoes, which could be interpreted as less effective pathogen interference. The effect was most striking on the 1% diet, where *P*. *gallinaceum* prevalence for both Tet and *w*Mel mosquitoes was near 100%. This increased prevalence could have been driven by starvation, similar to what is seen with *Plasmodium* infection in mosquitoes that experience larval nutritional stress [55, 62]. These data suggest that there are certain nutritional states or biological conditions that favour *Plasmodium* infection to the point where an inhibitory effect by *Wolbachia* is not possible. The fact that we also observed less effective interference on the 20% diet indicated that our results could not be solely explained by a starvation effect, and could have been due to a broader modulatory effect of host nutrition. Changing nutritional status also increased the median oocyst count for both Tet and *w*Mel mosquitoes, particularly on the 1% and 20% diets, however there was still a statistically significant effect of *w*Mel infection for the latter. Interestingly, *w*Mel limited the increase in the intensity of infection on the 1% and 5% diets, suggesting that the interference effects of *w*Mel at the intensity level occurred with the change in host nutritional status.

We observed greater overall *P*. *gallinaceum* intensity, and a different effect of *Wolbachia* on *P*. *gallinaceum* prevalence in one experiment. Infection with *P*. *gallinaceum* is typically subject to high variability, with great differences in prevalence and pathogen levels resulting from mosquito, parasite and avian genetic factors, and environmental factors [33, 63, 64]. Each experiment involved different chickens, with different genetic and immune responses that could have influenced the course of infection [65]. Across the three experiments, a stronger pathogen interference effect was associated with higher parasitemia, with no effect of *Wolbachia* observed in the experiment with the lowest parasitemia. While we did observe some variation between experiments, our results did suggest that host nutritional status can alter the response of *w*Mel to *P*. *gallinaceum* under some conditions, but also that this interference does not occur under all experimental conditions, and may only be induced during more severe infection.

In contrast, we saw no effect of host nutrition on DENV interference as no *w*Mel mosquitoes became infected on any diet across two experiments. This indicated that DENV interference is not affected by the change in host nutritional status, starvation or dietary excess. Furthermore, the different response to host nutritional status between the two pathogens suggests that there are potentially host biological factors that differentially affect interference against *P*. *gallinaceum* and DENV.

### Effect on processes associated with pathogen interference

We sought to determine if nutritional stress affected *Wolbachia* density, the expression of key immune genes and ROS levels, all of which have previously been linked to pathogen interference in either mosquitoes or *Drosophila*. These processes were characterized after mosquitoes fed on the different carbohydrate regimes for 7 days, the same time at which mosquitoes were infected with a pathogen in our experimental infection assays. These mosquitoes were not blood fed or infected with a pathogen in order to characterize basal changes due to diet and *Wolbachia*, and to avoid metabolic and transcriptional changes induced by blood feeding [56, 66].

High *Wolbachia* levels appear to be a key driver of pathogen interference [17, 67], and reduction of bacterial density can lead to weaker interference [68]. Critically, we saw no effect of diet on *Wolbachia* expression. This could potential indicate that the loss of interference against *P*. *gallinaceum* was not associated with a change in *Wolbachia* density. Although it is possible that such a change could occur in response to feeding on *Plasmodium*-infected blood, or that changes in *Wolbachia* levels at the tissue level led to a loss of bacterial density. An alternative explanation is that there was amelioration of another biological process linked to the phenotype.

Pathogen interference in mosquitoes has been strongly associated with the increased expression of key immune effector genes [22, 23, 38, 39]. We observed that high expression levels of four of these genes, *cece*, *defc*, *ctlga5* and *tsf* were consistently associated with *Wolbachia* infection for all diets. This could imply that a loss of immune gene activation did not underlie the less effective interference for *P*. *gallinaceum* that we observed on some diets, however it should be noted that we only measured basal immune gene levels, not in the context of *Plasmodium* infection, and this could potentially have led to different results. We did observe slight decreases in the expression of *tsf* on the 1% diet, and *defc* and *ctlga5* on the 20% diet in *w*Mel mosquitoes, and it is possible that our results could be explained by a similar effect across a large number of immune genes.

Similarly, we saw no effect of diet on the expression of regulatory genes in the IMD, JAK-STAT, JNK, and Toll mosquito immune pathways that might explain our results. Given that *Plasmodium* and DENV infections stimulate different immune pathways [69–73], it was possible that a diet-induced change in regulatory gene expression could stimulate higher infection levels. However, we only saw an effect of *Wolbachia* on the expression of *pias*, a negative regulator of the JAK-STAT pathway, and this change—higher expression in *w*Mel mosquitoes than Tet only for the 10% diet—did not adequately explain our results, as *w*Mel mosquitoes had similar *pias* levels across all diets. These results do not preclude an immune basis for the *Plasmodium*-specific response if it were to occur through genes or pathways other than those we measured.

Diet can influence levels of ROS and oxidative stress in insects [74, 75], and we observed a clear effect of mosquito diet on ROS induction, with equivalent H_2_O_2_ levels in *w*Mel and Tet mosquitoes from the 1% diet, and higher dietary carbohydrate levels associated with higher mean H_2_O_2_ levels in *w*Mel mosquitoes. In contrast, H_2_O_2_ levels in Tet mosquitoes were unaffected by diet, suggesting that there was a *Wolbachia*-specific interaction between nutritional and oxidative stress. The ROS induction phenotype is strongly correlated with pathogen interference in both mosquitoes and *Drosophila* [38, 40]. However, it is not universal amongst all host-strain associations where pathogen interference occurs, as is the case for *w*Mel-infected *Ae*. *albopictus*, where there is interference against DENV and Chikungunya virus infection [43, 76, 77]. The fact that ROS induction occurs for *w*Mel-infected *Ae*. *aegypti* suggests that its absence in *Ae*. *albopictus* is more likely due to the host mosquito than the *w*Mel strain, potentially because of the residual effects of co-adaptation with its native *Wolbachia* strains *w*AlbA and *w*AlbB.

The fact that loss of ROS induction occurred for the 1% diet, where *w*Mel and Tet mosquitoes has a similar susceptibility to *P*. *gallinaceum* infection is particularly interesting. ROS induction is part of the natural response to *Plasmodium* infection, with higher oxidative stress levels promoting parasite melanisation [78, 79]. Interestingly, levels of *tsf* in *w*Mel mosquitoes were also decreased for that diet. This gene is involved in iron transport and changes in its expression could have contributed to decreased ROS production and may be indicative of broader alterations to host oxidative stress response under conditions of starvation in *Wolbachia*-infected mosquitoes. Critically, as less effective *Plasmodium* interference, and high H_2_O_2_ levels were observed for the 20% diet, changes to ROS induction are unlikely to be the sole factor causing the differential effect of host nutrition that we observed on *Plasmodium* and DENV infection.

The stimulation of mitochondrial and oxidative stress gene expression by *Wolbachia* has been implicated in ROS induction, activation of the Toll immune pathway, and pathogen interference [22, 38, 39]. In *w*AlbB-infected *Ae*. *aegypti*, this effect was linked to a 23-fold increase in the expression of *duox-2*, which is thought to be an important enzyme for ROS production [38]. However in our experiments, and in *w*Mel-infected *Ae*. *albopictus*, *Wolbachia* did not affect *duox-2* levels, potentially because the gene lacks peroxidase activity, and therefore cannot directly stimulate ROS [43]. As we observed ROS induction without an effect of *Wolbachia* on *duox-2*, this implies that ROS induction occurs via a different process, potentially via the *duox-1* gene. Likewise, *duox-2* expression could not explain our ROS induction results, given the lack of an effect of host nutrition.

The enzyme *nos* is involved in the production of nitric oxide and reactive nitrogen species, and high *nos* levels have been linked to the inhibition of both *Plasmodium* and DENV in mosquitoes [80–82]. We observed no change in *nos* expression due to *Wolbachia*, indicating that this gene was unlikely to contribute to pathogen interference. Interestingly, we observed higher *nos* levels on the 1% and 5% diets for both *w*Mel and Tet mosquitoes, where the prevalence of *P*. *gallinaceum* infection was greater. This suggests that there is a link between nutritional stress and *nos* expression, and that *nos* levels can be induced under conditions of starvation without a strong effect on *P*. *gallinaceum* infection. It is possible that levels of H_2_O_2_, *nos* or the immune genes that we examined could have been changed in response to blood feeding or severity of *Plasmodium* infection, as both factors are linked to oxidative stress response [56, 66, 83, 84]. Additionally, there could have been systemic change in the mosquito oxidative stress and immune responses as a result of these processes, and this may have contributed to the response of *Wolbachia* to pathogens, even under conditions of starvation.

### Potential causes of diet-induced changes

We observed that changing host nutrition affected response to *Plasmodium* interference, ROS induction and *nos* expression. Furthermore there was differential fitness due to diet in the form of a longevity cost for low carbohydrate diets, which is not unexpected as dietary composition and insulin signalling affect lifespan in *Wolbachia*-infected insects [13, 85], and because *Wolbachia* increases the rate of resource depletion during starvation in larvae [86]. Starvation can stimulate immune response [53, 87, 88], as we observed with immune gene and *nos* expression on the 1% diet. It also reduces the availability of arginine and therefore affects levels of nitric oxide, and consequently affects the prevalence and intensity of *Plasmodium* infection [55, 89]. As such, it is possible that starvation-induced perturbations of the oxidative stress or nitric oxide response were the primary determining factor explaining our *Plasmodium* results from the 1% diet. Dietary excess is another form of nutritional stress, and in insects it causes obesity, alters the metabolism and biosynthesis of fats and carbohydrates, and alters oxidative stress response [90–93], which could explain some of the results for the 20% diet.

Metabolic interaction and competition for resources between the host and *Wolbachia* affects host gene expression, metabolic homeostasis, and physiological processes linked to metabolism [22, 39, 49, 60, 85, 94]. Resource competition leads to less effective pathogen interference in *D*. *melanogaster* [41, 60], and could underlie diet-based differences in *Plasmodium* interference, particularly on the 20% diet. Both *Plasmodium* and DENV exploit host carbohydrate metabolism [54, 95–97], and infection alters host carbohydrate homeostasis [57, 59, 98]. However, *Plasmodium* are probably more heavily reliant on host sugars, which they use for glycolysis, carbohydrate metabolism, and fatty acid II synthesis [99], and for development [95], and thus could be more highly affected by competition with *Wolbachia*. As the type of carbohydrate intake can influence susceptibility to *Plasmodium* infection in mosquitoes, there could be similar effects on the ability of *Wolbachia* to interfere with infection [95].

The composition of the host microbial community can be affected by host diet [100, 101], can alter host metabolic profile [102–104], and can affect response to pathogen infection [105–108]. Interestingly, the microbiota induce production of ROS, which can influence susceptibility to *Plasmodium* infection, and offers a potential explanation for the diet-induced changes we observed in oxidative stress response[109]. There is evidence of interaction between *Wolbachia* and the microbiota, in the form of mutual exclusion between *Wolbachia* and *Asaia* in anophelines [110], and a microbial influence on the vertical transmission of *Wolbachia* in transiently infected *An*. *stephensi* [111]. But the full extent of the interactions between *Wolbachia* and host microbiota are unclear, and there is certainly scope for a nutrition-driven interaction, that could affect a range of physiological processes including pathogen interference.

### Implications for pathogen interference

*w*Mel-infected mosquitoes have been present in the field for several years, where they maintain high levels of interference against different DENV isolates [52]. The issue of nutritional stress and pathogen interference is particularly important in the field where mosquitoes are released in locations with complex environmental and nutritional factors, and high levels of endemic dengue transmission [50, 112]. Adult *Ae*. *aegypti* nutritional needs are fulfilled by blood feeding when human hosts are available [113], and plant sugars when they are not. Recent work suggests that repeated blood feeding does not affect interference against DENV in *Ae*. *aegypti* [114]. While a sucrose-based diet is unlikely to be perfectly reflective of mosquito carbohydrate intake in the field, our diets did induce varying levels of nutritional stress, which could be similar to what mosquitoes in a heterogeneous environment might experience. What our results suggest is that DENV interference appears to be quite robust in the face of variable host nutritional status, and such an effect would be greatly beneficial if it were to occur in the field *Wolbachia*-infected mosquitoes. These data should be further clarified using different DENV serotypes, genetic isolates, and viral titres, as well as for other types of host diet, as these factors can all influence pathogen interference [25].

Our results did show that changes in host diet led to significantly weaker pathogen interference against *P*. *gallinaceum* under some host nutritional conditions, and that this may correspond to altered oxidative stress response. Yet because *Wolbachia*-infected *Ae*. *aegypti* are unlikely to become infected with *Plasmodium* in the field this does not represent a large issue for current control efforts. Potential problems could arise if a similar nutrition-based interaction were to occur in *Wolbachia*-infected anophelines. Critically, *P*. *gallinaceum* does not infect humans, and the effect we observed here may not occur for the mosquitoes and parasites responsible for human malaria, given their different immune and metabolic interactions [29, 30]. Pathogen interference has been observed against *P*. *falciparum* in *w*AlbB-infected *An*. *stephensi* [27], and future studies should determine the extent to which this phenotype is subject to environmental factors including nutrition, as this will have implications for future malaria control programs involving *Wolbachia*.

Perhaps the most interesting idea resulting from our data is the reinforcement and extension of the hypothesis of a complicated pathogen interference phenotype. Previous data indicates that the associated processes are not universal, with ROS induction being strain specific, and immune activation apparently specific to mosquitoes [37, 39, 43]. We have demonstrated that interference can also be pathogen specific, with diet-induced nutritional stress, and potentially starvation, affecting interference against *P*. *gallinaceum* but not DENV. It is also clear that host nutritional status can affect the ROS induction effect that has been linked to interference, and this should be further examined in the context of blood feeding, and experimental *Plasmodium* and DENV infection in order to characterize the effects of *Wolbachia* in a more natural nutritional state. These findings highlight the complicated nature of the phenotype, with the implication being that there is unlikely to be a ‘magic bullet’ explaining all occurrences of the phenotype. Rather, pathogen interference may arise through combinations of contributory factors with additive effects, and different pathways to interference occurring for different host-strain-pathogen combinations. The identity of these factors is currently unclear. However, given the breadth of *Wolbachia*’s effects on mosquito molecular biology, there are many potential candidates that have not yet been studied in great detail.

## Materials and Methods

### Mosquitoes and dietary manipulation

Two *Ae*. *aegypti* lines were used in these experiments. The first was infected with the *Wolbachia* strain *w*Mel (*w*Mel). This line was derived from the *w*Mel-transinfected line, originally generated in *Ae*. *aegypti* with an Australian genetic background [17]. The *w*Mel infection was introgressed into a Brazilian genetic background by breeding infected females with uninfected, field-collected males over nine generations, as previously described [112]. A subset of these mosquitoes were treated with tetracycline to clear the *Wolbachia* infection and then had their gut microbiota recolonised by introducing larval water from untreated mosquitoes into rearing trays, as previously described, with this line serving as a *Wolbachia*-uninfected control line (Tet) [112]. 50 wildtype, *Wolbachia*-uninfected F_1_-F_2_ males were introduced into colony cages for both *w*Mel and Tet lines each generation, in order to limit the occurrence of inbreeding and genetic divergence between the lines. These mosquitoes were collected near Rio de Janeiro, Brazil, and reared under laboratory conditions until eclosion, as described below. No wildtype males were introduced into experimental cages. *w*Mel mosquitoes used in these experiments were from G_14_—G_29_ post introgression into the Brazilian genetic background. Tet mosquitoes were from G_10_—G_25_ post microbial recolonization.

All mosquitoes in these experiments were reared under standard laboratory conditions in a climate-controlled insectary (temperature—27 ± 1°C, RH -70 ± 10%, photoperiod—12 hours light: dark). Mosquito larvae were hatched in 3L RO water containing ½ of a tetramin tropical tablet (Tetramin) ground into powder. Larval density was reduced to 50 per litre 24 hours after hatching. Larvae were then fed ½ a tetramin tropical tablet as required, with food levels equating to 1mg of food per larva per day. Pupae were sexed, collected and moved to small cylindrical cages (diameter– 16cm, height– 18cm) for experiments, with a maximum adult density of 150 per cage.

Adult mosquitoes were maintained on one of four different carbohydrate diets for the entirety of each experiment. The control diet was 10% sucrose, which was the same concentration provided to colony mosquitoes. The other three diets consisted of 1%, 5% and 20% sugar solution, with each inducing dietary stress either through starvation or excess. All diets were prepared by dissolving raw, granular cane sugar into RO water. Sucrose cups in experimental cages were changed every two days to prevent microbial contamination, with the solutions prepared fresh each time.

### DENV culture, infections and quantification

The virus used in these assays, DENV-3 MG20 (375) was originally isolated from infected patient blood in Brazil in 2012. The virus was cultured in C6-36 cells, titred using both the TCID-50 and plaque forming assay methods according to previously described methods [23]. Titre estimates were 10^10^–10^13^ infectious units/mL and 1.9x10^6^ infectious units/mL, respectively. Viral aliquots were stored at -80°C until the day of feeding. Cages of approximately 60 female mosquitoes were reared on carbohydrate diets as described above, and were starved overnight prior to feeding. Virus was mixed with freshly drawn blood from a willing volunteer at a 1:1 ratio. Blood used for feeding was screened for dengue virus using the Dengue NS1 Ag Strip Test (BioRad Laboratories). Mosquitoes were fed through glass feeders with pig intestine, using a heated waterbath system at a temperature of 37°C for 1 hour. Afterwards, non-blood fed, and semi-fed mosquitoes were removed and carbohydrate diets were re-introduced to cages. Half of the cage was collected at 7 days post-infection, and the other half collected at 14 days post-infection. Two independent feeding experiments were performed, using different aliquots from the same batch of virus.

Whole mosquito samples were stored at -80°C, and total RNA was extracted using the TRIzol protocol (ThermoFisher Scientific cat 15596–026) according to manufacturer’s instructions. Mosquitoes were homogenized in 200μL TRIzol using a mini beadbeater (BioSpec products). Samples were quantified using a NanoDrop 2000 UV-Vis spectrophotometer (ThermoFisher Scientific), and 1μg of total RNA was used for first strand cDNA synthesis using the M-MLV reverse transcriptase assay according to manufacturer’s instructions (Promega cat: C118A). cDNAs were then diluted 1:10 in nuclease-free water and stored at -30°C. Absolute DENV levels were quantified in duplicate for each cDNA, using a TaqMan-based assay with primers and a probe generalized to all four DENV serotypes (S2 Table). Each reaction contained the following: 2.5μL of cDNA, 2.50μL of TaqMan Universal Master Mix (ThermoFisher Scientific cat: 4304437), 0.50μL each of forward and reverse primers (10μM), 0.25μL of DENV probe (10μM), and 3.75μL of nuclease-free water. For a standard curve, we utilised a cloned DENV fragment, as previously described [23]. Serial dilutions of this fragment were run in triplicate between the concentrations of 10^7^ and 10^3^ copies for each plate. The run profile was 10 mins to denature at 95°C, followed by 40 amplification/cycles of 15 sec at 95°C followed by 1 min at 60°C using a Viia 7 Real-Time PCR System (ThermoFisher Scientific). DENV copies per sample were normalised per 1μg of total RNA. 12–20 samples were quantified per treatment.

### *Plasmodium gallinaceum* stock and infections

The *Plasmodium gallinaceum* stock used in these experiments was a long-term laboratory line (Brumpt, 1937, strain 8A). Cultures were maintained in the laboratory stored in chicken blood at -80°C, and through regular passage in 1–2 week old *Gallus gallus* chicks. Chicks were obtained at 1–2 day olds from Rivelli Poultry Farms, Mateus Leme, MG, Brazil, and were maintained in the FIOCRUZ Animal Facility during the course of experiments. Chicks were infected with *P*. *gallinaceum* infected blood drawn from previously infected chickens by trained personnel. Blood parasitemia levels were monitored during the course of infection by counting infected cells in a Giemsa-stained blood smear, with the blood obtained through toenail clipping. In each experiment, approximately 70 female mosquitoes from each of the 8 treatments (4 diets x 2 *Wolbachia* infection statuses) were fed on the different carbohydrate diets for 7 days. Mosquitoes were starved overnight and then allowed to feed on the same chick for 15 minutes per cage, with cages fed in random order. Blood parasitemia levels in the chicks on the day of feeding varied between experiments (E_1_: 11.16%, E_2_: 35.38%, E_3_: 4.10%). *Plasmodium* stocks used in these experiments had been passaged a maximum of three times. Post-feeding, the appropriate diets were re-introduced to cages, and non-blood fed, and semi-fed mosquitoes were removed. There were no noticeable effects of *Plasmodium* feeding on mosquito survival. At 7–8 days post-blood feeding, midguts were dissected in sterile 1x PBS before staining in 2% mercurochrome for 10 mins. Oocysts were visualised and counted via light microscopy. Mosquito numbers ranged between 33–53 per treatment across the three experiments.

### *Wolbachia* and immune gene transcription assays

12 pairs of 7–8 day-old, female, *w*Mel and Tet mosquitoes were collected after 7 days on their respective diets. Paired samples were used to reduce within treatment variation. This corresponded to the time when the mosquitoes in the pathogen infection assays were infected with either *P*. *gallinaceum* or DENV, however samples in these experiments were not infected with a pathogen. RNA extractions and first strand cDNA synthesis were performed as described above. The levels of 14 immune-related genes were quantified for all samples, while *Wolbachia* expression levels were quantified for only the *w*Mel samples using the *wsp* gene (S3 Table). Primer sequences used in these assays were either designed using Primer 3 V0.4.0 (http://bioinfo.ut.ee/primer3-0.4.0/), or as previously described [22, 23, 38], (S2 Table). Prior to use in experiments, each primer pair was assayed for specificity by melt curve analysis, with all pairs displaying only one peak. Additionally, we assayed primer efficiency by examining amplification performance with dilutions of cDNA samples. All primer pairs had an efficiency of between 90–100% at the dilution used in the experiments described below.

The immune transcription assays comprised of three parts. The first was an examination of genes previously shown to be highly upregulated by *w*Mel infection in *Ae*. *aegypti* with an Australian genetic background [39]. Four genes were examined: cecropin e (*cece*), defensin c (*defc*), transferrin (*tsf*) and c-type lectin galactose binding 5 (*ctlga5*). The second looked at regulatory genes in 4 different mosquito immune pathways. Eight genes were examined: *rel2* and *caspar* from the IMD pathway, *domeless* and *pias* from the JAK/STAT pathway, *ap-1* and *jnk* from the JNK pathway, and *rel1a* and *cactus* from the Toll pathway. The third part looked at two genes linked to stress response in mosquitoes. These were *duox-2*, which is linked to oxidative stress, and nitric oxide synthase (*nos*), which is linked to stress and *Plasmodium* infection. All genes were quantified in duplicate relative to the host ribosomal protein S17 (*rps17*). Each reaction contained the following: 2.50μL of cDNA, 7.50μL of SYBR Green PCR Master Mix (ThermoFisher Scientific cat 4309155), 0.75μL each of forward and reverse primers (10μM), and 4.50μL of nuclease-free water. The run profile was the same as described above. Mean normalised expression values were calculated for each gene using Q-Gene [115].

### ROS quantification assays

17–22 pairs of 7–8 day-old, female, *w*Mel and Tet mosquitoes were collected after 7 days on their respective diets. H_2_O_2_ levels in these samples were quantified using the Amplex Red Hydrogen Peroxide/Peroxidase Assay Kit (ThermoFisher Scientific cat A22188). Samples were collected on ice and then immediately homogenized in 200μL of 1x reaction buffer, using a mini beadbeater (BioSpec products), and then centrifuged for 2 mins at 14,200 x g, at 4°C. 50μL the supernatant was used to run the H_2_O_2_ assay, according to manufacturers instructions. Assays were run in black Nunc MicroWell 96-well Optical Bottom plates (ThermoFisher Scientific), and quantified using a Synergy 2 Multi-Mode Reader (BioTek), with an excitation wavelength of 545nm and an emission wavelength of 590nm.

### Longevity assay

A longevity assay was conducted to provide a basic measurement of the effects of host nutritional status on the fitness of *w*Mel-infected *Ae*. *aegypti* mosquitoes. *w*Mel larvae were reared as described above, and then female pupae were sexed and transferred to experimental cages, separated by carbohydrate diet. Pupal cups were removed from cages after 48 hours, so that all mosquitoes shared a similar age and development time. There were 3 replicate cages per diet, each containing approximately 45 females. Survival was monitored daily for the duration of the experiment, with dead mosquitoes removed from cages. Cage positions were rotated daily in order to normalize environmental variance.

### Ethics Statement

Maintenance of chickens, infections with *P*. *gallinaceum* and feeding of mosquitoes were conducted according to protocols that were reviewed and approved by The Commission of Ethical Animal Use (CEUA)/ FIOCRUZ (License—LW 38/12). This complied with Brazilian law 11794/08 which governs the use of animals for scientific purposes and principles as dictated by the Brazilian Society of Science on Laboratory Animals (SBCAL), and The National Council of Animal Experimentation Control (CONCEA).

The human blood used in these experiments was drawn from one willing, adult volunteer by trained medical personnel, after obtaining written consent. This process was conducted according to established guidelines, and approved by The Committee for Ethics in Research (CEP)/ FIOCRUZ (License—CEP 732.621). Our use of human blood was in accordance with Brazilian laws 196/1996 and 01/1988, which govern human ethics issues in scientific research in compliance with the National Council of Ethics in Research (CONEP).

### Statistical Analysis

*P*. *gallinaceum* infection data were analysed in two components; prevalence and intensity of infection. Uninfected mosquitoes were not considered in intensity analyses. Prevalence data were compared using binomial regression, and oocyst data by binomial negative regression, as there was overdispersion within the data set [116, 117]. Due to the fact that different *P. gallinaceum*-infected chickens were used in each experimental infection, the three experiments were analysed independently.

The test variable in these analyses was either infection status or oocyst number, while explanatory variables in the models were *Wolbachia* infection status, and dummy variables considering the effect of each diet, compared to the control 10% diet. A general effect of diet was not considered in the model, as we believed that the effect would differ between diets. *Wolbachia* x diet interaction terms were included, however these were generally not significant, and the models fit the data better after they were excluded (S1 Table).

Pairwise comparisons of differences in prevalence of *Plasmodium* infection due to *Wolbachia* for individual diets were calculated using Fisher’s exact test. Pairwise comparisons of oocyst levels for each diet were made using Mann-Whitney U tests. DENV prevalence data were compared by treatment using Fisher’s exact test. Viral intensity data for Tet mosquitoes were compared using one-way ANOVA.

Longevity data were compared statistically using Cox Regression. Expression data for immune activation genes, immune pathway regulators, stress response genes, *wsp* levels, and H_2_O_2_ levels were compared independently using univariate general linear models. When significant effects were observed, interactions between treatments were compared post-hoc using student’s *t*-tests and then using a false discovery rate of 0.05 as a multiple test correction. Statistical tests were applied only if the data fit the underlying assumptions. Statistical analyses were performed using R, SPSS V17 (IBM) and Prism 6.0g (Graphpad). Figures were prepared using Prism V 6.0g, Microsoft PowerPoint for Mac 2011, and GIMP v 2.8.14.

### Gene Accession Numbers

From VectorBase (https://www.vectorbase.org) unless noted.

ap-1 (AAEL011650-RA), c-type lectin galactose binding 5 (AAEL005641-RA), cactus (AAEL000709-RB), caspar (AAEL003579), cecropin e (AAEL000611-RA), defensin c (AAEL003832-RA), domeless (AAEL012471-RA), duox-2 (AAEL007562-RA), jnk (AAEL008634-RA), nitric oxide synthase (AAEL009745-RA), pias (AAEL015099-RA), rel1a (AAEL007696-RA), rel2 (AAEL007624-RA), ribosomal protein S17 (AAEL004157), transferrin (AAEL015458-RA), wolbachia surface protein (GenBank accession: EU395833.1).

## Supporting Information

S1 Fig Longevity of *w*Mel-infected *Ae*. *aegypti* fed on different carbohydrate regimes. The average survival time (± s.e.m.) of *w*Mel-infected *Ae*. *aegypti* was monitored daily across three cages per diet, with each containing 32–46 female mosquitoes. Mosquitoes were maintained on either 1%, 5%, 10% or 20% sucrose diets throughout the experiment. Data were compared by Cox Regression.
(TIF)

S2 Fig The expression of genes regulating the IMD, JAK-STAT, JNK and Toll immune pathways in *w*Mel-infected *Ae*. *aegypti* fed on different carbohydrate regimes. Expression levels of the IMD pathway regulatory genes *rel 2* **(A)** and *caspar* **(B)**, the JAK-STAT pathway regulatory genes *domeless* **(C)** and *pias* **(D)**, the JNK pathway regulatory genes *ap-1* **(E)** and *jnk* **(F)**, and the Toll pathway regulatory genes *rel 1A* **(G)** and *cactus* **(H)** were quantified for Tet (black circles) or *w*Mel (green circles) mosquitoes fed one of four carbohydrate diets. Gene expression values were normalized against host *rps17* expression. Each circle represents one pair of mosquitoes, with 12 samples examined for each treatment. Solid black lines represent mean expression (± s.e.m.). *P* value: Student’s *t* test, * < 0.05.
(TIF)

S1 Table Statistical Output from *Plasmodium gallinaceum* infection experiments. (DOCX)

S2 Table List of Primers and Probes. (DOCX)

S3 Table Mean normalised expression data from RT-qPCR experiments. (XLSX)

## References

[1] Bourtzis K, Dobson SL, Xi Z, Rasgon JL, Calvitti M, Moreira LA, et al Harnessing mosquito-*Wolbachia* symbiosis for vector and disease control. Acta Trop. 2014;132 Suppl:S150–63. 10.1016/j.actatropica.2013.11.004 24252486

[2] McGraw EA, O'Neill SL. Beyond insecticides: new thinking on an ancient problem. Nat Rev Microbiol. 2013;11(3):181–93. 10.1038/nrmicro2968 23411863

[3] Zug R, Hammerstein P. Still a host of hosts for *Wolbachia*: analysis of recent data suggests that 40% of terrestrial arthropod species are infected. PLoS One. 2012;7(6):e38544 PubMed Central PMCID: PMCPMC3369835. 10.1371/journal.pone.0038544 22685581 PMC3369835

[4] Caragata EP, Dutra HL, Moreira LA. Exploiting intimate relationships: Controlling mosquito-transmitted disease with *Wolbachia*. Trends in Parasitology. 2016. 10.1016/j.pt.2015.10.011 26776329

[5] Zug R, Hammerstein P. Bad guys turned nice? A critical assessment of *Wolbachia* mutualisms in arthropod hosts. Biol Rev Camb Philos Soc. 2014;90(1):89–111. 10.1111/brv.12098 24618033

[6] Werren JH, Baldo L, Clark ME. *Wolbachia*: master manipulators of invertebrate biology. Nat Rev Microbiol. 2008;6(10):741–51. 10.1038/nrmicro1969 18794912

[7] Turelli M, Hoffmann AA. Rapid spread of an inherited incompatibility factor in California *Drosophila*. Nature. 1991;353(6343):440–2. 10.1038/353440a0 1896086

[8] Dedeine F, Bouletreau M, Vavre F. *Wolbachia* requirement for oogenesis: occurrence within the genus *Asobara* (Hymenoptera, Braconidae) and evidence for intraspecific variation in *A*. *tabida*. Heredity (Edinb). 2005;95(5):394–400. 16118660 10.1038/sj.hdy.6800739

[9] Caragata EP, Real KM, Zalucki MP, McGraw EA. *Wolbachia* infection increases recapture rate of field-released *Drosophila melanogaster*. Symbiosis. 2011;54(1):55–60.

[10] Furihata S, Hirata M, Matsumoto H, Hayakawa Y. Bacteria Endosymbiont, *Wolbachia*, Promotes Parasitism of Parasitoid Wasp *Asobara japonica*. PLoS One. 2015;10(10):e0140914 PubMed Central PMCID: PMCPMC4619603. 10.1371/journal.pone.0140914 26492411 PMC4619603

[11] Foster J, Ganatra M, Kamal I, Ware J, Makarova K, Ivanova N, et al The *Wolbachia* genome of *Brugia malayi*: endosymbiont evolution within a human pathogenic nematode. PLoS Biol. 2005;3(4):e121 PubMed Central PMCID: PMCPMC1069646. 10.1371/journal.pbio.0030121 15780005 PMC1069646

[12] Brownlie JC, Cass BN, Riegler M, Witsenburg JJ, Iturbe-Ormaetxe I, McGraw EA, et al Evidence for metabolic provisioning by a common invertebrate endosymbiont, *Wolbachia pipientis*, during periods of nutritional stress. PLoS Pathog. 2009;5(4):e1000368 PubMed Central PMCID: PMCPMC2657209. 10.1371/journal.ppat.1000368 19343208 PMC2657209

[13] Ponton F, Wilson K, Holmes A, Raubenheimer D, Robinson KL, Simpson SJ. Macronutrients mediate the functional relationship between *Drosophila* and *Wolbachia*. Proc Biol Sci. 2015;282(1800):20142029 PubMed Central PMCID: PMCPMC4298205. 10.1098/rspb.2014.2029 25520356 PMC4298205

[14] Serbus LR, White PM, Silva JP, Rabe A, Teixeira L, Albertson R, et al The impact of host diet on *Wolbachia* titer in *Drosophila*. PLoS Pathog. 2015;11(3):e1004777 PubMed Central PMCID: PMCPMC4380406. 10.1371/journal.ppat.1004777 25826386 PMC4380406

[15] Hughes GL, Rasgon JL. Transinfection: a method to investigate *Wolbachia*-host interactions and control arthropod-borne disease. Insect Mol Biol. 2014;23(2):141–51. PubMed Central PMCID: PMCPMC3949162. 10.1111/imb.12066 24329998 PMC3949162

[16] McMeniman CJ, Lane RV, Cass BN, Fong AWC, Sidhu M, Wang YF, et al Stable introduction of a life-shortening *Wolbachia* infection into the mosquito *Aedes aegypti*. Science. 2009;323(5910):141–4. 10.1126/science.1165326 19119237

[17] Walker T, Johnson PH, Moreira LA, Iturbe-Ormaetxe I, Frentiu FD, McMeniman CJ, et al A non-virulent *Wolbachia* infection blocks dengue transmission and rapidly invades *Aedes aegypti* populations Nature. 2011;476:450–5. 10.1038/nature10355 21866159

[18] Xi Z, Khoo CC, Dobson SL. *Wolbachia* establishment and invasion in an *Aedes aegypti* laboratory population. Science. 2005;310(5746):326–8. Epub 2005/10/15. 10.1126/science.1117607 16224027

[19] Hedges LM, Brownlie JC, O'Neill SL, Johnson KN. *Wolbachia* and virus protection in insects. Science. 2008;322(5902):702-. 10.1126/science.1162418 18974344

[20] Teixeira L, Ferreira A, Ashburner M. The bacterial symbiont *Wolbachia* induces resistance to RNA viral infections in *Drosophila melanogaster*. Plos Biology. 2008;6(12):2753–63. 10.1371/journal.pbio.1000002 PMC2605931 19222304

[21] Bian GW, Xu Y, Lu P, Xie Y, Xi ZY. The endosymbiotic bacterium *Wolbachia* induces resistance to dengue virus in *Aedes aegypti*. Plos Pathogens. 2010;6(4):e1000833 10.1371/journal.ppat.1000833 20368968 PMC2848556

[22] Kambris Z, Cook PE, Phuc HK, Sinkins SP. Immune activation by life-shortening *Wolbachia* and reduced filarial competence in mosquitoes. Science. 2009;326(5949):134–6. 10.1126/science.1177531 19797660 PMC2867033

[23] Moreira LA, Iturbe-Ormaetxe I, Jeffery JA, Lu GJ, Pyke AT, Hedges LM, et al A *Wolbachia* symbiont in *Aedes aegypti* limits infection with Dengue, Chikungunya, and *Plasmodium*. Cell. 2009;139(7):1268–78. 10.1016/j.cell.2009.11.042 20064373

[24] van den Hurk AF, Hall-Mendelin S, Pyke AT, Frentiu FD, McElroy K, Day A, et al Impact of *Wolbachia* on infection with chikungunya and yellow fever viruses in the mosquito vector *Aedes aegypti*. PLoS Negl Trop Dis. 2012;6(11):e1892 PubMed Central PMCID: PMCPMC3486898. 10.1371/journal.pntd.0001892 23133693 PMC3486898

[25] Ferguson NM, Kien DT, Clapham H, Aguas R, Trung VT, Chau TN, et al Modeling the impact on virus transmission of *Wolbachia*-mediated blocking of dengue virus infection of *Aedes aegypti*. Sci Transl Med. 2015;7(279):279ra37 PubMed Central PMCID: PMCPMC4390297. 10.1126/scitranslmed.3010370 25787763 PMC4390297

[26] Joubert DA, Walker T, Carrington LB, De Bruyne JT, Kien DH, Hoang Nle T, et al Establishment of a *Wolbachia* Superinfection in *Aedes aegypti* Mosquitoes as a Potential Approach for Future Resistance Management. PLoS Pathog. 2016;12(2):e1005434 PubMed Central PMCID: PMCPMC4758728. 10.1371/journal.ppat.1005434 26891349 PMC4758728

[27] Bian G, Joshi D, Dong Y, Lu P, Zhou G, Pan X, et al *Wolbachia* invades *Anopheles stephensi* populations and induces refractoriness to *Plasmodium* infection. Science. 2013;340(6133):748–51. 10.1126/science.1236192 23661760

[28] Lutz HL, Patterson BD, Kerbis Peterhans JC, Stanley WT, Webala PW, Gnoske TP, et al Diverse sampling of East African haemosporidians reveals chiropteran origin of malaria parasites in primates and rodents. Mol Phylogenet Evol. 2016;99:7–15. 10.1016/j.ympev.2016.03.004 26975691

[29] Molina-Cruz A, Lehmann T, Knockel J. Could culicine mosquitoes transmit human malaria? Trends Parasitol. 2013;29(11):530–7. 10.1016/j.pt.2013.09.003 24140295 PMC10987011

[30] Waterhouse RM, Wyder S, Zdobnov EM. The *Aedes aegypti* genome: a comparative perspective. Insect Mol Biol. 2008;17(1):1–8. 9. 10.1111/j.1365-2583.2008.00772.x 18237279

[31] Hughes GL, Vega-Rodriguez J, Xue P, Rasgon JL. *Wolbachia* strain *w*AlbB enhances infection by the rodent malaria parasite *Plasmodium berghei* in *Anopheles gambiae* mosquitoes. Appl Environ Microbiol. 2012;78(5):1491–5. PubMed Central PMCID: PMCPMC3294472. 10.1128/AEM.06751-11 22210220 PMC3294472

[32] Murdock CC, Blanford S, Hughes GL, Rasgon JL, Thomas MB. Temperature alters Plasmodium blocking by Wolbachia. Sci Rep. 2014;4:3932 PubMed Central PMCID: PMCPMC3909897. 10.1038/srep03932 24488176 PMC3909897

[33] Baton LA, Pacidonio EC, Goncalves DS, Moreira LA. *w*Flu: characterization and evaluation of a native *Wolbachia* from the mosquito *Aedes fluviatilis* as a potential vector control agent. PLoS One. 2013;8(3):e59619 PubMed Central PMCID: PMCPMC3608659. 10.1371/journal.pone.0059619 23555728 PMC3608659

[34] Zélé F, Nicot A, Berthomieu A, Weill M, Duron O, Rivero A. *Wolbachia* increases susceptibility to *Plasmodium* infection in a natural system. Proc Biol Sci. 2014;281(1779):20132837 PubMed Central PMCID: PMCPMC3924077. 10.1098/rspb.2013.2837 24500167 PMC3924077

[35] Zélé F, Nicot A, Duron O, Rivero A. Infection with Wolbachia protects mosquitoes against Plasmodium-induced mortality in a natural system. J Evol Biol. 2012;25(7):1243–52. 10.1111/j.1420-9101.2012.02519.x 22533729

[36] Dodson BL, Hughes GL, Paul O, Matacchiero AC, Kramer LD, Rasgon JL. *Wolbachia* enhances West Nile virus (WNV) infection in the mosquito *Culex tarsalis*. PLoS Negl Trop Dis. 2014;8(7):e2965 PubMed Central PMCID: PMCPMC4091933. 10.1371/journal.pntd.0002965 25010200 PMC4091933

[37] Martinez J, Longdon B, Bauer S, Chan YS, Miller WJ, Bourtzis K, et al Symbionts commonly provide broad spectrum resistance to viruses in insects: a comparative analysis of *Wolbachia* strains. PLoS Pathog. 2014;10(9):e1004369 PubMed Central PMCID: PMCPMC4169468. 10.1371/journal.ppat.1004369 25233341 PMC4169468

[38] Pan X, Zhou G, Wu J, Bian G, Lu P, Raikhel AS, et al *Wolbachia* induces reactive oxygen species (ROS)-dependent activation of the Toll pathway to control dengue virus in the mosquito *Aedes aegypti*. Proceedings of the National Academy of Sciences of the United States of America. 2012;109(1):E23–E31. 10.1073/pnas.1116932108 22123956 PMC3252928

[39] Rancès E, Ye YH, Woolfit M, McGraw EA, O'Neill SL. The relative importance of innate immune priming in *Wolbachia*-mediated Dengue interference. Plos Pathogens. 2012;8(2). 10.1371/journal.ppat.1002548 PMC3285598 22383881

[40] Wong ZS, Brownlie JC, Johnson KN. Oxidative stress correlates with *Wolbachia*-mediated antiviral protection in *Wolbachia*-Drosophila associations. Appl Environ Microbiol. 2015;81(9):3001–5. PubMed Central PMCID: PMCPMC4393424. 10.1128/AEM.03847-14 25710364 PMC4393424

[41] Caragata EP, Rancès E, Hedges LM, Gofton AW, Johnson KN, O'Neill SL, et al Dietary cholesterol modulates pathogen blocking by *Wolbachia*. Plos Pathogens. 2013;9(6):e1003459 Epub 2013/07/05. PubMed Central PMCID: PMC3694857. 10.1371/journal.ppat.1003459 23825950 PMC3694857

[42] Chrostek E, Marialva MS, Yamada R, O'Neill SL, Teixeira L. High anti-viral protection without immune upregulation after interspecies *Wolbachia* transfer. PLoS One. 2014;9(6):e99025 PubMed Central PMCID: PMCPMC4049622. 10.1371/journal.pone.0099025 24911519 PMC4049622

[43] Molloy JC, Sinkins SP. *Wolbachia* Do Not Induce Reactive Oxygen Species-Dependent Immune Pathway Activation in *Aedes albopictus*. Viruses. 2015;7(8):4624–39. PubMed Central PMCID: PMCPMC4576197. 10.3390/v7082836 26287231 PMC4576197

[44] Hoffmann AA, Montgomery BL, Popovici J, Iturbe-Ormaetxe I, Johnson PH, Muzzi F, et al Successful establishment of *Wolbachia* in *Aedes* populations to suppress dengue transmission. Nature. 2011;476(7361):454–U107. 10.1038/nature10356 21866160

[45] Turelli M. Cytoplasmic incompatibility in populations with overlapping generations. Evolution. 2010;64(1):232–41. 10.1111/j.1558-5646.2009.00822.x 19686264

[46] Barton NH, Turelli M. Spatial waves of advance with bistable dynamics: cytoplasmic and genetic analogues of Allee effects. Am Nat. 2011;178(3):E48–75. 10.1086/661246 21828986

[47] Schraiber JG, Kaczmarczyk AN, Kwok R, Park M, Silverstein R, Rutaganira FU, et al Constraints on the use of lifespan-shortening *Wolbachia* to control dengue fever. J Theor Biol. 2012;297:26–32. 10.1016/j.jtbi.2011.12.006 22192469

[48] McMeniman CJ, O'Neill SL. A virulent *Wolbachia* infection decreases the viability of the Dengue vector *Aedes aegypti* during periods of embryonic quiescence. Plos Neglect Trop D. 2010;4(7):e748. 10.1371/journal.pntd.0000748 PMC2903475 20644622

[49] Turley AP, Moreira LA, O'Neill SL, McGraw EA. *Wolbachia* infection reduces blood-feeding success in the Dengue fever mosquito, *Aedes aegypti*. Plos Neglect Trop D. 2009;3(9):e516. 10.1371/journal.pntd.0000516 PMC2734393 19753103

[50] Nguyen TH, Nguyen HL, Nguyen TY, Vu SN, Tran ND, Le TN, et al Field evaluation of the establishment potential of *w*MelPop *Wolbachia* in Australia and Vietnam for dengue control. Parasit Vectors. 2015;8:563 PubMed Central PMCID: PMCPMC4625535. 10.1186/s13071-015-1174-x 26510523 PMC4625535

[51] Hoffmann AA, Iturbe-Ormaetxe I, Callahan AG, Phillips BL, Billington K, Axford JK, et al Stability of the *w*Mel *Wolbachia* Infection following invasion into *Aedes aegypti* populations. PLoS Negl Trop Dis. 2014;8(9):e3115 PubMed Central PMCID: PMCPMC4161343. 10.1371/journal.pntd.0003115 25211492 PMC4161343

[52] Frentiu FD, Zakir T, Walker T, Popovici J, Pyke AT, van den Hurk A, et al Limited dengue virus replication in field-collected *Aedes aegypti* mosquitoes infected with *Wolbachia*. PLoS Negl Trop Dis. 2014;8(2):e2688 Epub 2014/03/04. PubMed Central PMCID: PMC3930499. 10.1371/journal.pntd.0002688 24587459 PMC3930499

[53] Brown AE, Baumbach J, Cook PE, Ligoxygakis P. Short-term starvation of immune deficient *Drosophila* improves survival to gram-negative bacterial infections. PLoS One. 2009;4(2):e4490 PubMed Central PMCID: PMCPMC2637427. 10.1371/journal.pone.0004490 19221590 PMC2637427

[54] Liu K, Dong Y, Huang Y, Rasgon JL, Agre P. Impact of trehalose transporter knockdown on *Anopheles gambiae* stress adaptation and susceptibility to *Plasmodium falciparum* infection. Proc Natl Acad Sci U S A. 2013;110(43):17504–9. PubMed Central PMCID: PMCPMC3808592. 10.1073/pnas.1316709110 24101462 PMC3808592

[55] Takken W, Smallegange RC, Vigneau AJ, Johnston V, Brown M, Mordue-Luntz AJ, et al Larval nutrition differentially affects adult fitness and *Plasmodium* development in the malaria vectors *Anopheles gambiae* and *Anopheles stephensi*. Parasit Vectors. 2013;6(1):345 PubMed Central PMCID: PMCPMC4029273. 10.1186/1756-3305-6-345 24326030 PMC4029273

[56] Dana AN, Hillenmeyer ME, Lobo NF, Kern MK, Romans PA, Collins FH. Differential gene expression in abdomens of the malaria vector mosquito, *Anopheles gambiae*, after sugar feeding, blood feeding and *Plasmodium berghei* infection. BMC Genomics. 2006;7:119 PubMed Central PMCID: PMCPMC1508153. 10.1186/1471-2164-7-119 16712725 PMC1508153

[57] Fontaine KA, Sanchez EL, Camarda R, Lagunoff M. Dengue virus induces and requires glycolysis for optimal replication. J Virol. 2015;89(4):2358–66. PubMed Central PMCID: PMCPMC4338897. 10.1128/JVI.02309-14 25505078 PMC4338897

[58] Mayack C, Naug D. Parasitic infection leads to decline in hemolymph sugar levels in honeybee foragers. J Insect Physiol. 2010;56(11):1572–5. 10.1016/j.jinsphys.2010.05.016 20685210

[59] Nyasembe VO, Teal PE, Sawa P, Tumlinson JH, Borgemeister C, Torto B. *Plasmodium falciparum* infection increases *Anopheles gambiae* attraction to nectar sources and sugar uptake. Curr Biol. 2014;24(2):217–21. PubMed Central PMCID: PMCPMC3935215. 10.1016/j.cub.2013.12.022 24412210 PMC3935215

[60] Caragata EP, Rancès E, O'Neill SL, McGraw EA. Competition for amino acids between *Wolbachia* and the mosquito host, *Aedes aegypti*. Microb Ecol. 2014;67(1):205–18. 10.1007/s00248-013-0339-4 24337107

[61] Molloy JC, Sommer U, Viant MR, Sinkins SP. *Wolbachia* Modulates Lipid Metabolism in *Aedes albopictus* Mosquito Cells. Appl Environ Microbiol. 2016;82(10):3109–20. PubMed Central PMCID: PMCPMC4959074. 10.1128/AEM.00275-16 26994075 PMC4959074

[62] Vantaux A, Ouattarra I, Lefevre T, Dabire KR. Effects of larvicidal and larval nutritional stresses on *Anopheles gambiae* development, survival and competence for *Plasmodium falciparum*. Parasit Vectors. 2016;9(1):226. PubMed Central PMCID: PMCPMC4842262. 27107591 10.1186/s13071-016-1514-5 PMC4842262

[63] de Camargo MV, Consoli RA, Williams P, Krettli AU. Factors influencing the development of *Plasmodium gallinaceum* in *Aedes fluviatilis*. Mem Inst Oswaldo Cruz. 1983;78(1):83–94. 6645948 10.1590/s0074-02761983000100010

[64] Hume JC, Hamilton H 3rd, Lee KL, Lehmann T. Susceptibility of *Anopheles stephensi* to *Plasmodium gallinaceum*: a trait of the mosquito, the parasite, and the environment. PLoS One. 2011;6(6):e20156 PubMed Central PMCID: PMCPMC3111409. 10.1371/journal.pone.0020156 21694762 PMC3111409

[65] Macchi BM, Quaresma JA, Herculano AM, Crespo-Lopez ME, DaMatta RA, do Nascimento JL. Pathogenic action of *Plasmodium gallinaceum* in chickens: brain histology and nitric oxide production by blood monocyte-derived macrophages. Vet Parasitol. 2010;172(1–2):16–22. 10.1016/j.vetpar.2010.04.032 20537466

[66] Dana AN, Hong YS, Kern MK, Hillenmeyer ME, Harker BW, Lobo NF, et al Gene expression patterns associated with blood-feeding in the malaria mosquito *Anopheles gambiae*. BMC Genomics. 2005;6:5 PubMed Central PMCID: PMCPMC546002. 10.1186/1471-2164-6-5 15651988 PMC546002

[67] Martinez J, Ok S, Smith S, Snoeck K, Day JP, Jiggins FM. Should symbionts be nice or selfish? Antiviral effects of *Wolbachia* are costly but reproductive parasitism is not. PLoS Pathog. 2015;11(7):e1005021 PubMed Central PMCID: PMCPMC4488530. 10.1371/journal.ppat.1005021 26132467 PMC4488530

[68] Osborne SE, Iturbe-Ormaetxe I, Brownlie JC, O'Neill SL, Johnson KN. Antiviral protection and the importance of *Wolbachia* density and tissue tropism in *Drosophila simulans*. Appl Environ Microbiol. 2012;78(19):6922–9. PubMed Central PMCID: PMCPMC3457512. 10.1128/AEM.01727-12 22843518 PMC3457512

[69] Colpitts TM, Cox J, Vanlandingham DL, Feitosa FM, Cheng G, Kurscheid S, et al Alterations in the *Aedes aegypti* transcriptome during infection with West Nile, dengue and yellow fever viruses. PLoS Pathog. 2011;7(9):e1002189 PubMed Central PMCID: PMCPMC3164632. 10.1371/journal.ppat.1002189 21909258 PMC3164632

[70] Souza-Neto JA, Sim S, Dimopoulos G. An evolutionary conserved function of the JAK-STAT pathway in anti-dengue defense. Proc Natl Acad Sci U S A. 2009;106(42):17841–6. PubMed Central PMCID: PMCPMC2764916. 10.1073/pnas.0905006106 19805194 PMC2764916

[71] Xi Z, Ramirez JL, Dimopoulos G. The *Aedes aegypti* toll pathway controls dengue virus infection. PLoS Pathog. 2008;4(7):e1000098 PubMed Central PMCID: PMCPMC2435278. 10.1371/journal.ppat.1000098 18604274 PMC2435278

[72] Bahia AC, Kubota MS, Tempone AJ, Araujo HR, Guedes BA, Orfano AS, et al The JAK-STAT pathway controls *Plasmodium vivax* load in early stages of *Anopheles aquasalis* infection. PLoS Negl Trop Dis. 2011;5(11):e1317 PubMed Central PMCID: PMCPMC3206008. 10.1371/journal.pntd.0001317 22069502 PMC3206008

[73] Garver LS, Bahia AC, Das S, Souza-Neto JA, Shiao J, Dong Y, et al *Anopheles* Imd pathway factors and effectors in infection intensity-dependent anti-*Plasmodium* action. PLoS Pathog. 2012;8(6):e1002737 PubMed Central PMCID: PMCPMC3369948. 10.1371/journal.ppat.1002737 22685401 PMC3369948

[74] Burger JM, Hwangbo DS, Corby-Harris V, Promislow DE. The functional costs and benefits of dietary restriction in *Drosophila*. Aging Cell. 2007;6(1):63–71. 10.1111/j.1474-9726.2006.00261.x 17266676

[75] Rovenko BM, Kubrak OI, Gospodaryov DV, Perkhulyn NV, Yurkevych IS, Sanz A, et al High sucrose consumption promotes obesity whereas its low consumption induces oxidative stress in *Drosophila melanogaster*. J Insect Physiol. 2015;79:42–54. 10.1016/j.jinsphys.2015.05.007 26050918

[76] Blagrove MS, Arias-Goeta C, Di Genua C, Failloux AB, Sinkins SP. A *Wolbachia w*Mel transinfection in *Aedes albopictus* is not detrimental to host fitness and inhibits Chikungunya virus. PLoS Negl Trop Dis. 2013;7(3):e2152 PubMed Central PMCID: PMCPMC3610642. 10.1371/journal.pntd.0002152 23556030 PMC3610642

[77] Blagrove MS, Arias-Goeta C, Failloux AB, Sinkins SP. *Wolbachia* strain *w*Mel induces cytoplasmic incompatibility and blocks dengue transmission in *Aedes albopictus*. Proc Natl Acad Sci U S A. 2012;109(1):255–60. PubMed Central PMCID: PMCPMC3252941. 10.1073/pnas.1112021108 22123944 PMC3252941

[78] Kumar S, Christophides GK, Cantera R, Charles B, Han YS, Meister S, et al The role of reactive oxygen species on *Plasmodium* melanotic encapsulation in *Anopheles gambiae*. Proc Natl Acad Sci U S A. 2003;100(24):14139–44. PubMed Central PMCID: PMCPMC283559. 10.1073/pnas.2036262100 14623973 PMC283559

[79] Molina-Cruz A, DeJong RJ, Charles B, Gupta L, Kumar S, Jaramillo-Gutierrez G, et al Reactive oxygen species modulate *Anopheles gambiae* immunity against bacteria and *Plasmodium*. J Biol Chem. 2008;283(6):3217–23. 10.1074/jbc.M705873200 18065421

[80] Luckhart S, Vodovotz Y, Cui L, Rosenberg R. The mosquito *Anopheles stephensi* limits malaria parasite development with inducible synthesis of nitric oxide. Proc Natl Acad Sci U S A. 1998;95(10):5700–5. PubMed Central PMCID: PMCPMC20442. 9576947 10.1073/pnas.95.10.5700 PMC20442

[81] Peterson TM, Gow AJ, Luckhart S. Nitric oxide metabolites induced in *Anopheles stephensi* control malaria parasite infection. Free Radic Biol Med. 2007;42(1):132–42. PubMed Central PMCID: PMCPMC1764505. 10.1016/j.freeradbiomed.2006.10.037 17157200 PMC1764505

[82] Ramos-Castaneda J, Gonzalez C, Jimenez MA, Duran J, Hernandez-Martinez S, Rodriguez MH, et al Effect of nitric oxide on Dengue virus replication in *Aedes aegypti* and *Anopheles albimanus*. Intervirology. 2008;51(5):335–41. 10.1159/000175639 19023217

[83] Oliveira JH, Goncalves RL, Lara FA, Dias FA, Gandara AC, Menna-Barreto RF, et al Blood meal-derived heme decreases ROS levels in the midgut of *Aedes aegypti* and allows proliferation of intestinal microbiota. PLoS Pathog. 2011;7(3):e1001320 PubMed Central PMCID: PMCPMC3060171. 10.1371/journal.ppat.1001320 21445237 PMC3060171

[84] Upton LM, Povelones M, Christophides GK. *Anopheles gambiae* blood feeding initiates an anticipatory defense response to *Plasmodium berghei*. J Innate Immun. 2015;7(1):74–86. PubMed Central PMCID: PMCPMC4564949. 10.1159/000365331 25247883 PMC4564949

[85] Ikeya T, Broughton S, Alic N, Grandison R, Partridge L. The endosymbiont *Wolbachia* increases insulin/IGF-like signalling in *Drosophila*. Proc Biol Sci. 2009;276(1674):3799–807. PubMed Central PMCID: PMCPMC2817276. 10.1098/rspb.2009.0778 19692410 PMC2817276

[86] Ross PA, Endersby NM, Hoffmann AA. Costs of Three *Wolbachia* Infections on the Survival of *Aedes aegypti* Larvae under Starvation Conditions. PLoS Negl Trop Dis. 2016;10(1):e0004320 PubMed Central PMCID: PMCPMC4706305. 10.1371/journal.pntd.0004320 26745630 PMC4706305

[87] Becker T, Loch G, Beyer M, Zinke I, Aschenbrenner AC, Carrera P, et al FOXO-dependent regulation of innate immune homeostasis. Nature. 2010;463(7279):369–73. 10.1038/nature08698 20090753

[88] Scott RC, Schuldiner O, Neufeld TP. Role and regulation of starvation-induced autophagy in the *Drosophila* fat body. Dev Cell. 2004;7(2):167–78. 10.1016/j.devcel.2004.07.009 15296714

[89] Telang A, Qayum AA, Parker A, Sacchetta BR, Byrnes GR. Larval nutritional stress affects vector immune traits in adult yellow fever mosquito *Aedes aegypti* (*Stegomyia aegypti*). Med Vet Entomol. 2012;26(3):271–81. 10.1111/j.1365-2915.2011.00993.x 22112201

[90] Al Saud SN, Summerfield AC, Alic N. Ablation of insulin-producing cells prevents obesity but not premature mortality caused by a high-sugar diet in *Drosophila*. Proc Biol Sci. 2015;282(1800):20141720 PubMed Central PMCID: PMCPMC4298201. 10.1098/rspb.2014.1720 25520354 PMC4298201

[91] Morris SN, Coogan C, Chamseddin K, Fernandez-Kim SO, Kolli S, Keller JN, et al Development of diet-induced insulin resistance in adult *Drosophila melanogaster*. Biochim Biophys Acta. 2012;1822(8):1230–7. PubMed Central PMCID: PMCPMC3601833. 10.1016/j.bbadis.2012.04.012 22542511 PMC3601833

[92] Musselman LP, Fink JL, Narzinski K, Ramachandran PV, Hathiramani SS, Cagan RL, et al A high-sugar diet produces obesity and insulin resistance in wild-type *Drosophila*. Dis Model Mech. 2011;4(6):842–9. PubMed Central PMCID: PMCPMC3209653. 10.1242/dmm.007948 21719444 PMC3209653

[93] Musselman LP, Fink JL, Ramachandran PV, Patterson BW, Okunade AL, Maier E, et al Role of fat body lipogenesis in protection against the effects of caloric overload in *Drosophila*. J Biol Chem. 2013;288(12):8028–42. PubMed Central PMCID: PMCPMC3605622. 10.1074/jbc.M112.371047 23355467 PMC3605622

[94] McMeniman CJ, Hughes GL, O'Neill SL. A *Wolbachia* symbiont in *Aedes aegypti* disrupts mosquito egg development to a greater extent when mosquitoes feed on nonhuman versus human blood. J Med Entomol. 2011;48(1):76–84. 21337952 10.1603/me09188

[95] Weathersby AB, Noblet R. *Plasmodium gallinaceum*: development in *Aedes aegypti* maintained on various carbohydrate diets. Exp Parasitol. 1973;34(3):426–31. 4773579 10.1016/0014-4894(73)90102-1

[96] Toledo KA, Fermino ML, Andrade Cdel C, Riul TB, Alves RT, Muller VD, et al Galectin-1 exerts inhibitory effects during DENV-1 infection. PLoS One. 2014;9(11):e112474 PubMed Central PMCID: PMCPMC4231055. 10.1371/journal.pone.0112474 25392933 PMC4231055

[97] Wichit S, Jittmittraphap A, Hidari KI, Thaisomboonsuk B, Petmitr S, Ubol S, et al Dengue virus type 2 recognizes the carbohydrate moiety of neutral glycosphingolipids in mammalian and mosquito cells. Microbiol Immunol. 2011;55(2):135–40. 10.1111/j.1348-0421.2010.00293.x 21265875

[98] Zhao YO, Kurscheid S, Zhang Y, Liu L, Zhang L, Loeliger K, et al Enhanced survival of *Plasmodium*-infected mosquitoes during starvation. PLoS One. 2012;7(7):e40556 PubMed Central PMCID: PMCPMC3393683. 10.1371/journal.pone.0040556 22808193 PMC3393683

[99] Gardner MJ, Hall N, Fung E, White O, Berriman M, Hyman RW, et al Genome sequence of the human malaria parasite *Plasmodium falciparum*. Nature. 2002;419(6906):498–511. PubMed Central PMCID: PMCPMC3836256. 10.1038/nature01097 12368864 PMC3836256

[100] Colman DR, Toolson EC, Takacs-Vesbach CD. Do diet and taxonomy influence insect gut bacterial communities? Mol Ecol. 2012;21(20):5124–37. 10.1111/j.1365-294X.2012.05752.x 22978555

[101] Dillon RJ, Webster G, Weightman AJ, Keith Charnley A. Diversity of gut microbiota increases with aging and starvation in the desert locust. Antonie Van Leeuwenhoek. 2010;97(1):69–77. 10.1007/s10482-009-9389-5 19876756

[102] Chen S, Bagdasarian M, Walker ED. *Elizabethkingia anophelis*: molecular manipulation and interactions with mosquito hosts. Appl Environ Microbiol. 2015;81(6):2233–43. PubMed Central PMCID: PMCPMC4345385. 10.1128/AEM.03733-14 25595771 PMC4345385

[103] Ridley EV, Wong AC, Westmiller S, Douglas AE. Impact of the resident microbiota on the nutritional phenotype of *Drosophila melanogaster*. PLoS One. 2012;7(5):e36765 PubMed Central PMCID: PMCPMC3346728. 10.1371/journal.pone.0036765 22586494 PMC3346728

[104] Wong AC, Dobson AJ, Douglas AE. Gut microbiota dictates the metabolic response of *Drosophila* to diet. J Exp Biol. 2014;217(Pt 11):1894–901. PubMed Central PMCID: PMCPMC4037322. 10.1242/jeb.101725 24577449 PMC4037322

[105] Carissimo G, Pondeville E, McFarlane M, Dietrich I, Mitri C, Bischoff E, et al Antiviral immunity of *Anopheles gambiae* is highly compartmentalized, with distinct roles for RNA interference and gut microbiota. Proc Natl Acad Sci U S A. 2015;112(2):E176–85. PubMed Central PMCID: PMCPMC4299212. 10.1073/pnas.1412984112 25548172 PMC4299212

[106] Dong Y, Manfredini F, Dimopoulos G. Implication of the mosquito midgut microbiota in the defense against malaria parasites. PLoS Pathog. 2009;5(5):e1000423 PubMed Central PMCID: PMCPMC2673032. 10.1371/journal.ppat.1000423 19424427 PMC2673032

[107] Gendrin M, Rodgers FH, Yerbanga RS, Ouedraogo JB, Basanez MG, Cohuet A, et al Antibiotics in ingested human blood affect the mosquito microbiota and capacity to transmit malaria. Nat Commun. 2015;6:5921 PubMed Central PMCID: PMCPMC4338536. 10.1038/ncomms6921 25562286 PMC4338536

[108] Ramirez JL, Short SM, Bahia AC, Saraiva RG, Dong Y, Kang S, et al *Chromobacterium Csp_P* reduces malaria and dengue infection in vector mosquitoes and has entomopathogenic and *in vitro* anti-pathogen activities. PLoS Pathog. 2014;10(10):e1004398 PubMed Central PMCID: PMCPMC4207801. 10.1371/journal.ppat.1004398 25340821 PMC4207801

[109] Gonçalves RL, Oliveira JH, Oliveira GA, Andersen JF, Oliveira MF, Oliveira PL, et al Mitochondrial reactive oxygen species modulate mosquito susceptibility to *Plasmodium* infection. PLoS One. 2012;7(7):e41083 PubMed Central PMCID: PMCPMC3399787. 10.1371/journal.pone.0041083 22815925 PMC3399787

[110] Rossi P, Ricci I, Cappelli A, Damiani C, Ulissi U, Mancini MV, et al Mutual exclusion of *Asaia* and *Wolbachia* in the reproductive organs of mosquito vectors. Parasit Vectors. 2015;8:278 PubMed Central PMCID: PMCPMC4445530. 10.1186/s13071-015-0888-0 25981386 PMC4445530

[111] Hughes GL, Dodson BL, Johnson RM, Murdock CC, Tsujimoto H, Suzuki Y, et al Native microbiome impedes vertical transmission of *Wolbachia* in *Anopheles* mosquitoes. Proc Natl Acad Sci U S A. 2014;111(34):12498–503. PubMed Central PMCID: PMCPMC4151774. 10.1073/pnas.1408888111 25114252 PMC4151774

[112] Dutra HL, Dos Santos LM, Caragata EP, Silva JB, Villela DA, Maciel-de-Freitas R, et al From lab to field: the influence of urban landscapes on the invasive potential of *Wolbachia* in Brazilian *Aedes aegypti* mosquitoes. PLoS Negl Trop Dis. 2015;9(4):e0003689 PubMed Central PMCID: PMCPMC4408005. 10.1371/journal.pntd.0003689 25905888 PMC4408005

[113] Edman JD, Strickman D, Kittayapong P, Scott TW. Female *Aedes aegypti* (Diptera: *Culicidae*) in Thailand rarely feed on sugar. J Med Entomol. 1992;29(6):1035–8. 1460619 10.1093/jmedent/29.6.1035

[114] Amuzu HE, Simmons CP, McGraw EA. Effect of repeat human blood feeding on *Wolbachia* density and dengue virus infection in *Aedes aegypti*. Parasit Vectors. 2015;8:246 PubMed Central PMCID: PMCPMC4413987. 10.1186/s13071-015-0853-y 25903749 PMC4413987

[115] Simon P. Q-Gene: processing quantitative real-time RT-PCR data. Bioinformatics. 2003;19(11):1439–40. 12874059 10.1093/bioinformatics/btg157

[116] Dobson AJ, Barnett AG. An Introduction to Generalized Linear Models. 3. ed. ed. Boca Raton: Chapman and Hall/CRC; 2008.

[117] Triola MF. Introdução à Estatística. 7. ed. ed. Rio de Janeiro: LTC; 1999.

