# Supplementary figures and images for: Diet-Induced Nutritional Stress and Pathogen Interference in Wolbachia-Infected Aedes aegypti
Source: PLoS Negl Trop Dis. 2016 Nov 28;10(11):e0005158. doi: 10.1371/journal.pntd.0005158 (PMC5125575; doi:10.1371/journal.pntd.0005158)

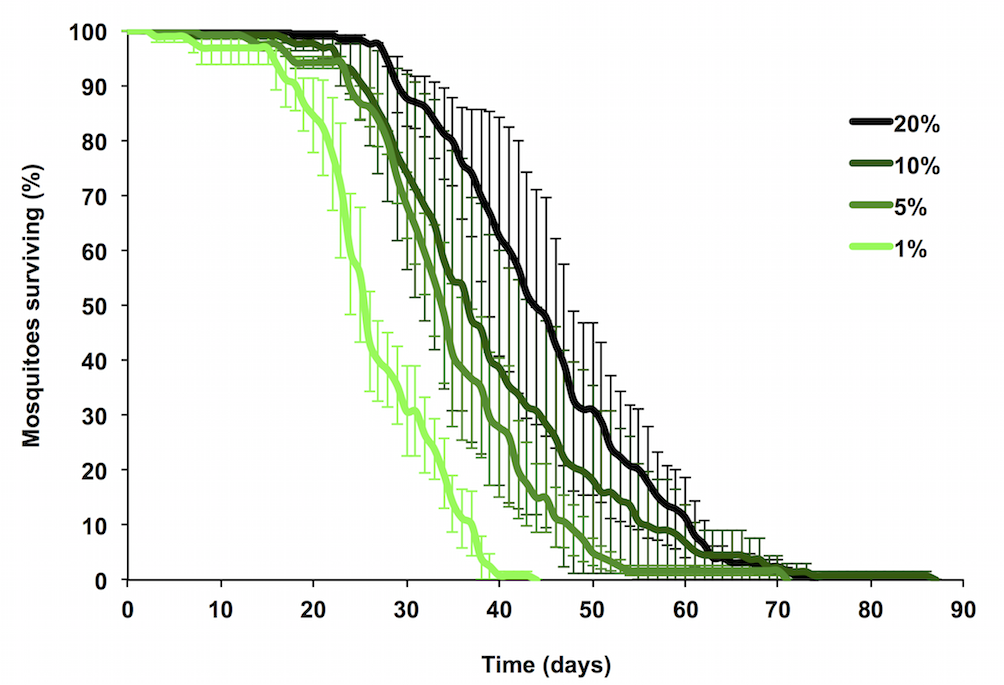

Supplement: S1 Fig — The average survival time (± s.e.m.) of wMel-infected Ae. aegypti was monitored daily across three cages per diet, with each containing 32–46 female mosquitoes. Mosquitoes were maintained on either 1%, 5%, 10% or 20% sucrose diets throughout the experiment. Data were compared by Cox Regression. (TIF) [file pntd.0005158.s001.tif]

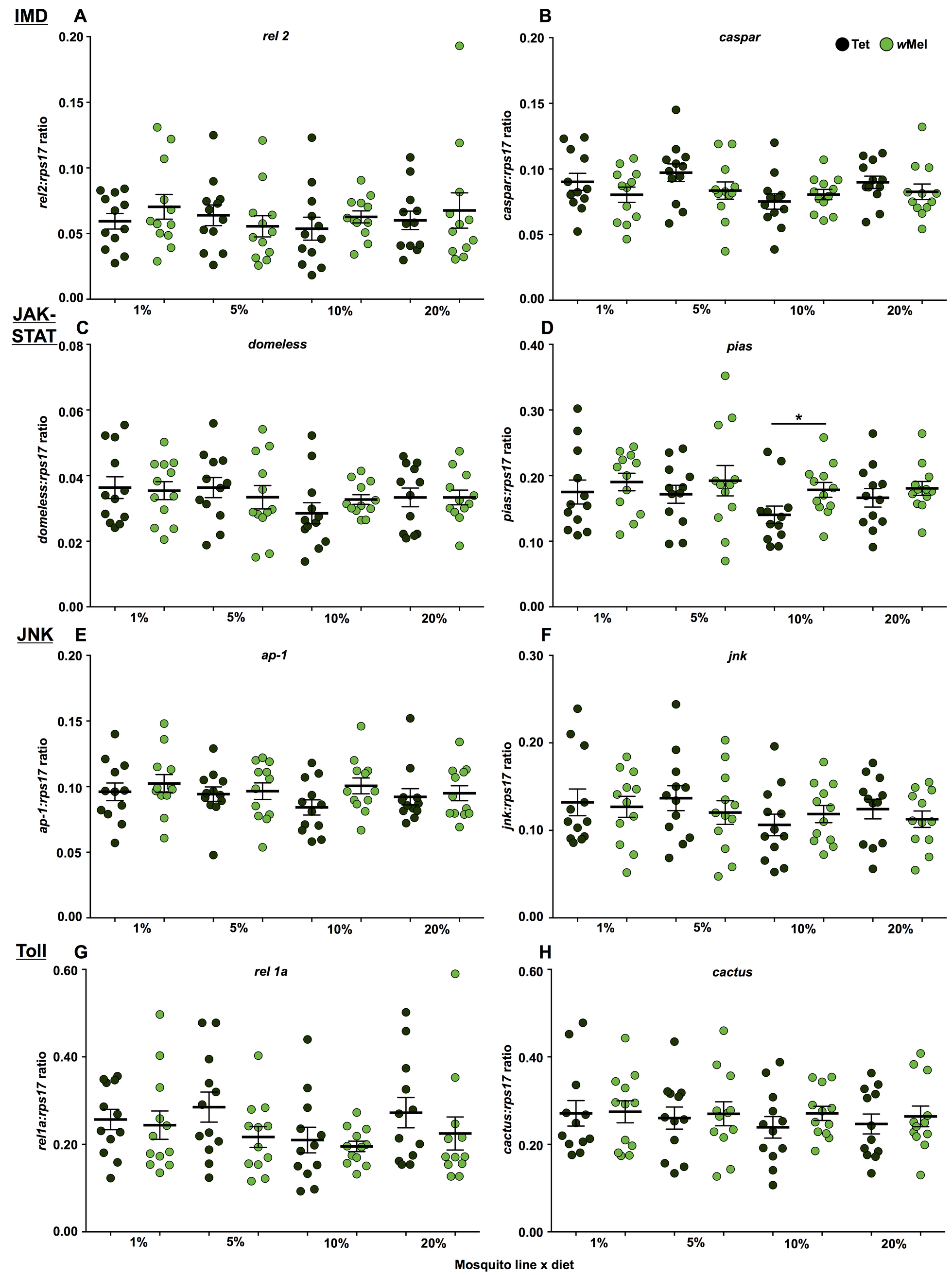

Supplement: S2 Fig — Expression levels of the IMD pathway regulatory genes rel 2 (A) and caspar (B), the JAK-STAT pathway regulatory genes domeless (C) and pias (D), the JNK pathway regulatory genes ap-1 (E) and jnk (F), and the Toll pathway regulatory genes rel 1A (G) and cactus (H) were quantified for Tet (black circles) or wMel (green circles) mosquitoes fed one of four carbohydrate diets. Gene expression values were normalized against host rps17 expression. Each circle represents one pair of mosquitoes, with 12 samples examined for each treatment. Solid black lines represent mean expression (± s.e.m.). P value: Student’s t test, * < 0.05. (TIF) [file pntd.0005158.s002.tif]
