# Supplementary material for: Diet-Induced Nutritional Stress and Pathogen Interference in Wolbachia-Infected Aedes aegypti
Source: PLoS Negl Trop Dis. 2016 Nov 28;10(11):e0005158. doi: 10.1371/journal.pntd.0005158 (PMC5125575; doi:10.1371/journal.pntd.0005158)
Supplement: S1 Table — (DOCX) [file pntd.0005158.s003.docx]

**S1 Table: Output from statistical analyses used to compare *Plasmodium gallinaceum* prevalence and intensity data in the main text.**

Significant terms are highlighted in red.

**Experiment One**

**Binomial Regression (Prevalence)**

|  | **Estimate** | **Std. Error** | ***Z* value** | ***P*** |
| --- | --- | --- | --- | --- |
| **Intercept** | 0.4498 | 0.2930 | 1.535 | 0.1247 |
| ***Wolbachia*** | -1.0031 | 0.3139 | -3.195 | 0.0014 |
| **Diet - 1%** | 3.7476 | 0.7617 | 4.920 | 8.64e-07 |
| **Diet - 5%** | 0.4200 | 0.3429 | 1.225 | 0.2206 |
| **Diet - 20%** | 2.6251 | 0.4687 | 5.600 | 2.14e-08 |

**Fisher’s Exact Tests (Prevalence)**

|  | **Tet Infected** | **Mel Infected** | ***P*** |
| --- | --- | --- | --- |
| **1%** | 32/34 | 38/38 | 0.2195 |
| **5%** | 30/42 | 15/33 | 0.0326 |
| **10%** | 21/34 | 15/41 | 0.0381 |
| **20%** | 42/42 | 39/46 | 0.0127 |

**Binomial Negative Regression (Intensity)**

|  | **Estimate** | **Std. Error** | ***Z* value** | ***P*** |
| --- | --- | --- | --- | --- |
| **Intercept** | 1.2930 | 0.1933 | 6.688 | 2.26e-11 |
| ***Wolbachia*** | -0.2520 | 0.1615 | -1.560 | 0.119 |
| **Diet - 1%** | 2.5900 | 0.2381 | 10.876 | < 2e-16 |
| **Diet - 5%** | 0.9409 | 0.2391 | 3.936 | 8.29e-05 |
| **Diet - 20%** | 2.5156 | 0.2278 | 11.041 | < 2e-16 |

**Mann Whitney U tests (Intensity)**

|  | **Tet med** | **Mel med** | ***U*** | ***P*** |
| --- | --- | --- | --- | --- |
| **1%** | 41.50 | 17.50 | 403 | 0.0151 |
| **5%** | 8.50 | 9.00 | 207 | 0.6717 |
| **10%** | 3.50 | 2.00 | 149 | 0.9865 |
| **20%** | 38.50 | 23.00 | 691.5 | 0.2303 |

**Experiment Two**

**Binomial Regression (Prevalence)**

|  | **Estimate** | **Std. Error** | ***Z* value** | ***P*** |
| --- | --- | --- | --- | --- |
| **Intercept** | 0.3315 | 0.2470 | 1.342 | 0.18 |
| ***Wolbachia*** | -1.8033 | 0.2695 | -6.691 | 2.21e-11 |
| **Diet - 1%** | 3.5169 | 0.4715 | 7.458 | 8.76e-14 |
| **Diet - 5%** | 1.7535 | 0.3364 | 5.212 | 1.87e-07 |
| **Diet - 20%** | 1.2649 | 0.3245 | 3.898 | 9.69e-05 |

**Fisher’s Exact Tests (Prevalence)**

|  | **Tet Infected** | **Mel Infected** | ***P*** |
| --- | --- | --- | --- |
| **1%** | 47/50 | 48/52 | 1.0000 |
| **5%** | 46/51 | 29/52 | 0.0001 |
| **10%** | 31/50 | 8/53 | <0.0001 |
| **20%** | 41/50 | 23/50 | 0.0003 |

**Binomial Negative Regression (Intensity)**

|  | **Estimate** | **Std. Error** | ***Z* value** | ***P*** |
| --- | --- | --- | --- | --- |
| **Intercept** | 1.4988 | 0.1762 | 8.508 | < 2e-16 |
| ***Wolbachia*** | -1.4021 | 0.1529 | -9.168 | < 2e-16 |
| **Diet - 1%** | 2.2382 | 0.2195 | 10.195 | < 2e-16 |
| **Diet - 5%** | 1.2544 | 0.2211 | 5.672 | 1.41e-08 |
| **Diet - 20%** | 1.4727 | 0.2220 | 6.635 | 3.25e-11 |

**Mann Whitney U tests (Intensity)**

|  | **Tet med** | **Mel med** | ***U*** | ***P*** |
| --- | --- | --- | --- | --- |
| **1%** | 36.00 | 6.00 | 284.5 | <0.0001 |
| **5%** | 14.00 | 4.00 | 291 | <0.0001 |
| **10%** | 3.00 | 4.50 | 112 | 0.6860 |
| **20%** | 10.00 | 7.00 | 389.5 | 0.2530 |

**Experiment Three**

**Binomial Regression (Prevalence)**

|  | **Estimate** | **Std. Error** | ***Z* value** | ***P*** |
| --- | --- | --- | --- | --- |
| **Intercept** | 0.1570 | 0.2412 | 0.651 | 0.515230 |
| ***Wolbachia*** | 0.1136 | 0.2472 | 0.459 | 0.646066 |
| **Diet - 1%** | 0.6851 | 0.3320 | 2.063 | 0.039078 |
| **Diet - 5%** | 1.4639 | 0.3576 | 4.094 | 4.25e-05 |
| **Diet - 20%** | 1.0788 | 0.3268 | 3.301 | 0.000964 |

**Fisher’s Exact Tests (Prevalence)**

|  | **Tet Infected** | **Mel Infected** | ***P*** |
| --- | --- | --- | --- |
| **1%** | 26/31 | 26/42 | 0.0659 |
| **5%** | 34/43 | 41/46 | 0.2487 |
| **10%** | 25/47 | 27/47 | 0.8358 |
| **20%** | 32/44 | 41/49 | 0.2176 |

**Binomial Negative Regression (Intensity)**

|  | **Estimate** | **Std. Error** | ***Z* value** | ***P*** |
| --- | --- | --- | --- | --- |
| **Intercept** | 2.3315 | 0.1801 | 12.947 | < 2e-16 |
| ***Wolbachia*** | -0.1403 | 0.1654 | -0.849 | 0.396071 |
| **Diet - 1%** | 0.9967 | 0.2406 | 4.142 | 3.44e-05 |
| **Diet - 5%** | 0.8044 | 0.2282 | 3.525 | 0.000423 |
| **Diet - 20%** | 0.3662 | 0.2263 | 1.618 | 0.105607 |

**Mann Whitney U tests (Intensity)**

|  | **Tet med** | **Mel med** | ***U*** | ***P*** |
| --- | --- | --- | --- | --- |
| **1%** | 41.00 | 22.00 | 214.5 | 0.0231 |
| **5%** | 31.50 | 18.00 | 502 | 0.0376 |
| **10%** | 12.00 | 10.00 | 285 | 0.3410 |
| **20%** | 10.00 | 14.00 | 517 | 0.1229 |

**Initial iterations of prevalence/intensity models**

These models included Wolbachia x diet interactions for the three test diets, however these proved to be poor explanatory factors, and were subsequently removed from the models described above, and in the main text.

Experiment 1

Response: infected/uninfected - prevalence

Explanatory: wolbachia, diet, model = binomial

Coefficients:

Estimate Std. Error z value Pr(>|z|)

(Intercept) 0.3567 0.3485 1.024 0.30604

wolbachia[T.1] -0.8267 0.4793 -1.725 0.08459 .

diet[T.Diet_1%] 2.4159 0.8079 2.990 0.00279 **

diet[T.Diet_5%] 0.5596 0.4880 1.147 0.25143

diet[T.Diet_20%] 18.2094 1006.4650 0.018 0.98557

wolbachia[T.1]:diet[T.Diet_1%] 16.6202 1058.1121 0.016 0.98747

wolbachia[T.1]:diet[T.Diet_5%] -0.2719 0.6846 -0.397 0.69120

wolbachia[T.1]:diet[T.Diet_20%] -16.0217 1006.4652 -0.016 0.98730

AIC: 264.22

Response: oocysts - intensity

Explanatory: wolbachia, diet model=binomial negative

Coefficients:

Estimate Std. Error z value Pr(>|z|)

(Intercept) 1.23581 0.25296 4.885 1.03e-06 ***

wolbachia[T.1] -0.14578 0.34776 -0.419 0.67506

diet[T.Diet_1%] 2.71656 0.34640 7.842 4.42e-15 ***

diet[T.Diet_5%] 1.00287 0.33377 3.005 0.00266 **

diet[T.Diet_20%] 2.54946 0.33077 7.708 1.28e-14 ***

wolbachia[T.1]:diet[T.Diet_1%] -0.24226 0.47675 -0.508 0.61135

wolbachia[T.1]:diet[T.Diet_5%] -0.11713 0.47925 -0.244 0.80691

wolbachia[T.1]:diet[T.Diet_20%] -0.06202 0.45601 -0.136 0.89181

AIC: 2286.9

Experiment 2

Response: infected/uninfected - prevalence

Explanatory: wolbachia, diet, model = binomial

Coefficients:

Estimate Std. Error z value Pr(>|z|)

(Intercept) 0.4895 0.2914 1.680 0.092913 .

wolbachia[T.1] -2.2168 0.4818 -4.601 4.2e-06 ***

diet[T.Diet_1%] 2.2620 0.6629 3.412 0.000645 ***

diet[T.Diet_5%] 1.7297 0.5537 3.124 0.001787 **

diet[T.Diet_20%] 1.0268 0.4695 2.187 0.028728 *

wolbachia[T.1]:diet[T.Diet_1%] 1.9501 0.9260 2.106 0.035213 *

wolbachia[T.1]:diet[T.Diet_5%] 0.2294 0.7293 0.315 0.753124

wolbachia[T.1]:diet[T.Diet_20%] 0.5401 0.6694 0.807 0.419792

AIC: 398.53

Response: oocysts - intensity

Explanatory: wolbachia, diet model=binomial negative

Coefficients:

Estimate Std. Error z value Pr(>|z|)

(Intercept) 1.5041 0.2176 6.913 4.75e-12 ***

wolbachia[T.1] -1.4139 0.3241 -4.362 1.29e-05 ***

diet[T.Diet_1%] 2.3362 0.3011 7.759 8.58e-15 ***

diet[T.Diet_5%] 1.3587 0.3009 4.516 6.30e-06 ***

diet[T.Diet_20%] 1.2026 0.3026 3.974 7.06e-05 ***

wolbachia[T.1]:diet[T.Diet_1%] -0.2059 0.4378 -0.470 0.638

wolbachia[T.1]:diet[T.Diet_5%] -0.2296 0.4418 -0.520 0.603

wolbachia[T.1]:diet[T.Diet_20%] 0.4990 0.4422 1.128 0.259

AIC: 2470.6

Experiment 3

Response: infected/uninfected - prevalence

Explanatory: wolbachia, diet, model = binomial

Coefficients:

Estimate Std. Error z value Pr(>|z|)

(Intercept) 0.1278 0.2923 0.437 0.66190

wolbachia[T.1] 0.1723 0.4153 0.415 0.67830

diet[T.Diet_1%] 1.5208 0.5691 2.672 0.00754 **

diet[T.Diet_5%] 1.2013 0.4754 2.527 0.01150 *

diet[T.Diet_20%] 0.8530 0.4473 1.907 0.05650 .

wolbachia[T.1]:diet[T.Diet_1%] -1.3354 0.7155 -1.866 0.06198 .

wolbachia[T.1]:diet[T.Diet_5%] 0.6027 0.7331 0.822 0.41096

wolbachia[T.1]:diet[T.Diet_20%] 0.4810 0.6607 0.728 0.46655

AIC: 399.21

Response: oocysts - intensity

Explanatory: wolbachia, diet model=binomial negative

Coefficients:

Estimate Std. Error z value Pr(>|z|)

(Intercept) 2.36045 0.22311 10.580 < 2e-16 ***

wolbachia[T.1] -0.19933 0.31622 -0.630 0.528460

diet[T.Diet_1%] 1.26432 0.35080 3.604 0.000313 ***

diet[T.Diet_5%] 0.85002 0.32082 2.649 0.008061 **

diet[T.Diet_20%] -0.09964 0.32120 -0.310 0.756410

wolbachia[T.1]:diet[T.Diet_1%] -0.53242 0.47755 -1.115 0.264890

wolbachia[T.1]:diet[T.Diet_5%] -0.09102 0.45069 -0.202 0.839956

wolbachia[T.1]:diet[T.Diet_20%] 0.77293 0.44767 1.727 0.084247 .

AIC: 2548.1
