# Supplementary material for: Diet-Induced Nutritional Stress and Pathogen Interference in Wolbachia-Infected Aedes aegypti
Source: PLoS Negl Trop Dis. 2016 Nov 28;10(11):e0005158. doi: 10.1371/journal.pntd.0005158 (PMC5125575; doi:10.1371/journal.pntd.0005158)
Supplement: S2 Table — (DOCX) [file pntd.0005158.s004.docx]

**S2 Table – Primers and Probes**

Primers were designed for this study using Primer 3 unless specified.

**AP-1 - AAEL011650-RA**

F - 5’-TGCCATCAAGAACAACGTGG-3’

R - 5’-ATACCCGTTTGTCCAGCTGA-3’

Amplicon size: 93bp

**C-type Lectin - Galactose Binding (CTLGA5) - AAEL005641-RA (Kambris et al, 2009)**

F - 5’-GTCTCCGGGTGCAATACACT-3’

R - 5’-CCCTATCGTTCCACTTCCAA-3’

Amplicon size: 123bp

**Cactus - AAEL000709-RB (Xi et al, 2008)**

F - 5’-AGACAGCCGCACCTTCGATTCC-3’

R - 5’-CGCTTCGGTAGCCTCGTGGATC-3’

Amplicon size: 232bp

**Caspar - AAEL003579-RA (Xi et al, 2008)**

F - 5’-GAATCCGAGCGAGCCGATGC-3’

R - 5’-CGTAGTCCAGCGTTGTGAGGTC-3’

Amplicon size: 271bp

**Cecropin E (CECE) AAEL000611-RA (Kambris et al, 2009)**

F - 5’-TTGCACTCGTTCTGCTCATC-3’

R - 5’-ACACGTTTTCCGACTCCTTC-3’

Amplicon size: 103bp

**Defensin C (DEFC) AAEL003832-RA (Xi et al, 2008)**

F - 5’-TTGTTTGCTTCGTTGCTCTTT-3’

R - 5’-ATCTCCTACACCGAACCCACT-3’

Amplicon size: 199bp

**Dengue Virus (Rancès et al, 2012)**

F - 5’-AAGGACTAGAGGTTAGAGGAGACCC-3’

R - 5’-CGTTCTGTGCCTGGAATGATG-3’

Probe - 5’-**ABY**-AACAGCATATTGACGCTGGGAGAGACCAGA-**BHQ1**-3’

Amplicon size: 108bp

**Domeless - AAEL012471-RA (Moreira et al, 2009)**

F - 5’-AAGATGTTCGTAACGACTCGGTCATT-3’

R - 5’-GGTGAGATTGTACGTAACATGATCGGTAT-3’

Amplicon size: 149bp

**Dual Oxidase 2 (DUOX-2) AAEL007562-RA (Pan et al, 2012)**

F - 5’-CGTGGCGGAGTATTTCAGT-3'

R - 5’-GCAAATTCATCAGCAACCAC-3'

Amplicon size: 125bp

**JNK - AAEL008634-RA**

F - 5’-CAACCGCCTCAAAGCTAGTC-3’

R - 5’-CACGTTGATGTAGCTGTGCA-3’

Amplicon size: 112bp

**Nitric Oxide Synthase (NOS) AAEL009745-RA**

F - 5’-TCTTCGCCCAGGATCTGTAC-3’

R - 5’-GATTTCGACGAGGCAGCAAT-3’

Amplicon size: 91bp

**PIAS - AAEL015099-RA (Souza-Neto et al, 2009)**

F - 5’-GCTGCAACGCATGAAAACTA-3’

R - 5’-CAGACGGGACAGTTCCAAGT-3’

Amplicon size: 252bp

**Rel 1A - AAEL007696-RA (Xi et al, 2008)**

F - 5’-TGGTGGTGGTGTCCTGCGTAAC-3’

R - 5’-CTGCCTGGCGTGACCGTATCC-3’

Amplicon size: 242bp

**Rel 2 - AAEL007624-RA**

F - 5’-TGAATGTGCTGTTGGGTCAT-3’

R – 5’-TTTTTACACATCACCGCCAA-3’

Amplicon size: 100bp

**Ribosomal protein S17 (RpS17) AAEL004157 (Cook et al, 2006)**

F - 5’-CACTCCCAGGTCCGTGGTAT-3’

R - 5’-GGACACTTCCGGCACGTAGT-3’

Amplicon size: 81bp

**Transferrin AAEL015458-RA (Kambris et al, 2009)**

F - 5’-GTTCCGGTACAACCTGGAGA-3’

R - 5’-TTCAGCTCGATCAGGGAAGT-3’

Amplicon size: 116bp

**Wolbachia surface protein (WSP) (Caragata et al, 2013)**

F - 5’-TGGTCCAATAAGTGATGAAGAAAC-3’

R - 5’-AAAAATTAAACGCTACTCCA-3’

Amplicon size: 632bp

**References**

Caragata EP, Rancès E, Hedges LM, Gofton AW, Johnson KN, O'Neill SL, et al. Dietary cholesterol modulates pathogen blocking by *Wolbachia*. Plos Pathogens. 2013;9(6):e1003459. Epub 2013/07/05. doi: 10.1371/journal.ppat.1003459. PubMed PMID: 23825950; PubMed Central PMCID: PMC3694857.

Cook PE, Hugo LE, Iturbe-Ormaetxe I, Williams, CR, Chenoweth, SF, Ritchie, SA, et al. The use of transcriptional profiles to predict adult mosquito age under field conditions. Proceedings of the National Academy of Sciences of the United States of America. 2006;103(48):18060-18065. Doi 10.1073/pnas.0604875103.

Kambris Z, Cook PE, Phuc HK, Sinkins SP. Immune activation by life-shortening *Wolbachia* and reduced filarial competence in mosquitoes. Science. 2009;326(5949):134-6. doi: Doi 10.1126/Science.1177531. PubMed PMID: ISI:000270355600053.

Moreira LA, Iturbe-Ormaetxe I, Jeffery JA, Lu GJ, Pyke AT, Hedges LM, et al. A *Wolbachia* symbiont in *Aedes aegypti* limits infection with Dengue, Chikungunya, and *Plasmodium*. Cell. 2009;139(7):1268-78. doi: Doi 10.1016/J.Cell.2009.11.042. PubMed PMID: ISI:000273048700015.

Pan X, Zhou G, Wu J, Bian G, Lu P, Raikhel AS, et al. *Wolbachia* induces reactive oxygen species (ROS)-dependent activation of the Toll pathway to control dengue virus in the mosquito *Aedes aegypti*. Proceedings of the National Academy of Sciences of the United States of America. 2012;109(1):E23-E31. doi: 10.1073/pnas.1116932108. PubMed PMID: WOS:000298876500005.

Rancès E, Ye YH, Woolfit M, McGraw EA, O'Neill SL. The relative importance of innate immune priming in *Wolbachia*-mediated Dengue interference. Plos Pathogens. 2012;8(2). doi: e1002548.

Souza-Neto JA, Sim S, Dimopoulos G. An evolutionary conserved function of the JAK-STAT pathway in anti-dengue defense. Proc Natl Acad Sci U S A. 2009;106(42):17841-6. doi: 10.1073/pnas.0905006106. PubMed PMID: 19805194; PubMed Central PMCID: PMCPMC2764916.

Xi Z, Ramirez JL, Dimopoulos G. The *Aedes aegypti* toll pathway controls dengue virus infection. PLoS Pathog. 2008;4(7):e1000098. doi: 10.1371/journal.ppat.1000098. PubMed PMID: 18604274; PubMed Central PMCID: PMCPMC2435278.
